# An Integrated Proteomic and Transcriptomic Analysis Reveals the Venom Complexity of the Bullet Ant *Paraponera clavata*

**DOI:** 10.3390/toxins12050324

**Published:** 2020-05-14

**Authors:** Samira R. Aili, Axel Touchard, Regan Hayward, Samuel D. Robinson, Sandy S. Pineda, Hadrien Lalagüe, Mrinalini, Irina Vetter, Eivind A. B. Undheim, R. Manjunatha Kini, Pierre Escoubas, Matthew P. Padula, Garry S. A. Myers, Graham M. Nicholson

**Affiliations:** 1 School of Life Sciences, University of Technology Sydney, Broadway, NSW 2007, Australia; Samira.Aili@uts.edu.au (S.R.A.); Regan.Hayward@student.uts.edu.au (R.H.); Matthew.Padula@uts.edu.au (M.P.P.); Garry.Myers@uts.edu.au (G.S.A.M.); 2 CNRS, UMR Ecologie des Forêts de Guyane, AgroParisTech, CIRAD, INRA, Université de Guyane, Université des Antilles, Campus Agronomique, BP316, 97379 Kourou cedex, France; axel.touchard2@gmail.com (A.T.); hadrien.lalague@ecofog.gf (H.L.); 3 Institute for Molecular Bioscience, The University of Queensland, St. Lucia, QLD 4072, Australia; sam.robinson@uq.edu.au (S.D.R.); i.vetter@imb.uq.edu.au (I.V.); 4 Centre for Advanced Imaging, The University of Queensland, St. Lucia, QLD 4072, Australia; e.undheim@uq.edu.au; 5 Brain and Mind Centre, University of Sydney, Camperdown, Sydney, NSW 2050, Australia; sandy.pineda@sydney.edu.au; 6 Protein Science Laboratory, Department of Biological Science, Faculty of Science, National University of Singapore, Singapore 117543, Singapore; dbsmrin@nus.edu.sg (M.); dbskinim@nus.edu.sg (R.M.K.); 7 Department of Pharmacology, Yong Loo Lin School of Medicine, National University of Singapore, Singapore 117600, Singapore; 8 Centre for Biodiversity Dynamics, Department of Biology, Norwegian University of Science and Technology, 7491 Trondheim, Norway; 9 Centre for Ecological and Evolutionary Synthesis, Department of Biosciences, University of Oslo, PO Box 1066 Blindern, 0316 Oslo, Norway; 10 Faculty of Science, University of Nice, 06000 Nice, France; escoubas@venometech.com

**Keywords:** neurotoxins, phospholipases, hyaluronidase, RP-HPLC, DRG neurons, paraponeritoxin

## Abstract

A critical hurdle in ant venom proteomic investigations is the lack of databases to comprehensively and specifically identify the sequence and function of venom proteins and peptides. To resolve this, we used venom gland transcriptomics to generate a sequence database that was used to assign the tandem mass spectrometry (MS) fragmentation spectra of venom peptides and proteins to specific transcripts. This was performed alongside a shotgun liquid chromatography–mass spectrometry (LC-MS/MS) analysis of the venom to confirm that these assigned transcripts were expressed as proteins. Through the combined transcriptomic and proteomic investigation of *Paraponera clavata* venom, we identified four times the number of proteins previously identified using 2D-PAGE alone. In addition to this, by mining the transcriptomic data, we identified several novel peptide sequences for future pharmacological investigations, some of which conform with inhibitor cysteine knot motifs. These types of peptides have the potential to be developed into pharmaceutical or bioinsecticide peptides.

## 1. Introduction

Animal venoms are an increasingly popular source of drug leads, as they are rich in bioactive peptides. These peptides have evolved over millions of years, and they target a wide variety of receptors and other biological processes [1,2]. Currently there are six venom-derived peptides on the therapeutic drug market and one insecticide [2]. However, there is potential for many more, as only a small fraction of venom-derived peptides has been investigated. New developments in sensitivity and accuracy in mass spectrometry and transcriptomic sequencing are likely to accelerate this process. One of the hurdles of venom proteomics investigations has been the lack of appropriate databases to assign peptide sequence information using bottom-up mass spectrometry. The use of venom gland mRNA to deduce protein sequence information is becoming increasingly common, particularly in conjunction with venom proteomics analysis, as this overcomes some of the problems of predicting open reading frames (ORFs) or *de novo* assembly. 

Transcriptomics is a useful tool for *de novo* generation of comprehensive venom gland mRNA profiles*,* and it requires only a small amount of tissue. Transcriptomic approaches are relatively unbiased as they capture almost all the diversity present in the venom gland at the time of tissue harvesting, as opposed to proteomics where ion suppression can significantly confound analyte ionisation and detection for certain types of instrumentation. However, using transcriptomics alone is insufficient. For example, transcriptomics involves prediction of the peptide/ protein sequence from the thousands of transcripts in the transcriptome, the majority of which do not encode for venom peptides/ protein, rather they are part of the cellular machinery [3]. Furthermore, post-translational modifications (PTMs), including proteolytic cleavage, are not detected by transcriptomics, unlike proteomic approaches. Combining proteomics and transcriptomics allows both synthesised proteins and PTMs to be identified and enables confirmation of the mature peptide/protein sequence. In addition, the transcriptome permits expression estimation for genes of interest.

In this study, we investigated the venom profile of *P. clavata* using an integrated proteomic/ transcriptomic approach. *Paraponera clavata* is a large ant species (>2 cm in length) found in the New World tropics and renowned for its potent and painful sting [4,5]. The venom of *P. clavata* has received significant attention at a proteomic level, with seven publications to date [4,5,6,7,8,9,10,11]. All of these previously published studies focused on poneratoxin, a 25-residue peptide, and the most abundant component of the venom. Poneratoxin elicits neurotoxic activity in cockroaches by prolonging action potentials and generating slow repetitive activity by inducing a voltage-activated sodium channel (Na_V_1.7) current with slowed inactivation at negative potentials [6,10]. Nevertheless, there are several other components in this venom that remain uncharacterised [12].

In this investigation, we identified novel components of *P. clavata* venom and highlight the advantages of using combined proteomics and transcriptomics approaches for a holistic overview of the complexity of ant venom arsenals.

## 2. Results

### 2.1. P. clavata Venom Gland Transcriptome Profile

In order to investigate the diversity and expression of venom toxins and toxin-like sequences in *P. clavata*, transcriptome sequencing was performed on venom glands and sacs. The transcriptome assembly was used as a database for exploring the *P. clavata* venom proteome. 

#### 2.1.1. Illumina Sequencing

Illumina Hiseq 2500 sequencing of the cDNA library from *P. clavata* venom glands yielded over 17 million paired end reads after quality control (QC). Briefly, trimmed reads were *de novo* assembled using Trinity [13], producing a total of 54,242 contigs. RSEM was used to determine expression levels of contigs from reads mapped using Bowtie using the transcripts per million (TPM) estimates. Assembly statistics are provided in Table 1.

#### 2.1.2. *P. clavata* Transcriptome Annotation

To identify the types of proteins and toxins expressed in the venom gland of *P. clavata*, the contig library was used to interrogate the NCBInr database using the BLASTx algorithm [14] with an e-value cut-off of e^−4^. The search generated 37,140 hits matching proteins in the database, which represent 68% of the transcriptome. Twenty of the top 35 hits were to other ant species and the remainder were to other hymenopteran species. The highest number of hits recorded were to the ant *Harpegnathos saltator* (Figure S1), reflecting the close phylogenetic relationship between these species. 

#### 2.1.3. Identification and Classification of *P. clavata* Toxins

**Figure 1:**
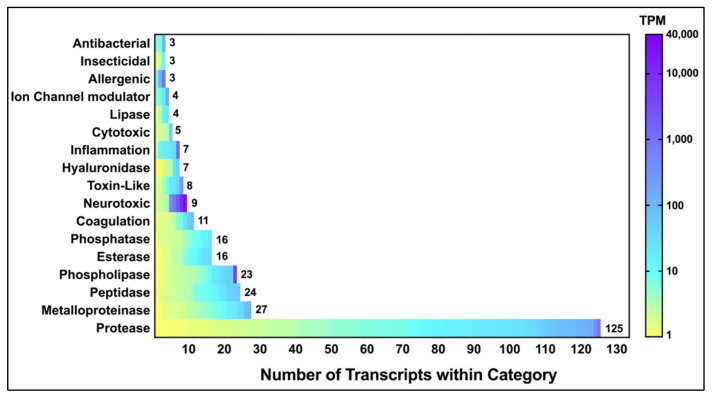
Abundance and expression levels of *P. clavata* toxin and toxin-like transcripts. The heat map shows contigs classified into 17 categories. Toxin expression levels are represented by transcript per million (TPM) and are depicted by a colour scale with yellow being least abundant (≥1 TPM) and purple signifying highest expressed (see right-hand scale). The *x*-axis indicates the number of transcripts within each category. The “Protease” category is comprised of serine and aspartic proteases and cathepsins.

We identified 354 toxin-like sequences that were classified into 17 different families (Figure 1). These were identified through a manual search of the BLASTx output using over 50 different keywords such as “phospholipase” and “toxin” (Table S1). A total of 17,102 contigs (32%) had no significant BLASTx hit. Figure 1 also includes hits from the output of the Tox|Blast module [15], which recovered other toxin-like transcripts not observed in the BLASTx output.

The venom gland transcriptome revealed that the majority of transcripts were proteases, e.g., serine proteases and metalloproteinases (Figure 1). However, neurotoxins were the most highly expressed contigs, based on TPM values. These were subsequently identified, through proteomic analyses, as poneratoxin isoforms (Section 2.3.1). Contigs in the “toxin-like” category included peptides such as ant “ω-conotoxin-like” proteins which, despite having an assigned mode of action to inhibit voltage-activated calcium channels, have not been proven to have any toxin activity. The full list of toxin and toxin-like sequences identified through BLASTx and Tox|Note can be found in Supplementary File S1.

Among the transcripts, a poneratoxin-encoding contig was the second most abundantly expressed at 39,657 TPM, with the structural protein actin being the most highly expressed protein (55,551 TPM). Other highly expressed toxin-like transcripts included phospholipases, serine proteases, arginine kinases and metalloproteinases. The remainder of the toxin-like transcripts had relatively lower TPM values such as defensin-2 (89 TPM) and cathepsin (77 TPM). The most highly expressed toxin and toxin-like transcripts, identified through BLASTx, are summarized in Table 2. All *P. clavata* venom gland contigs shown in Table 2 were subsequently named using the nomenclature proposed in the Methods section. 

Although the majority of hits from Tox|Note were to toxins with proven toxicity, several unusual hits were observed e.g., the 130 kDa toxin α-latrotoxin. α-Latrotoxin is composed of several ankyrin repeats and only the full-length protein, presently only detected in the venom of theridiid spiders, is neurotoxic [1,16]. Transcripts from *P. clavata* only matched to the ankyrin domains of the 130 kDa toxin, but not other regions important for activity. Ankyrin domains are also present in other non-toxic proteins [1,16]. Calglandulin was also identified by Tox|Note as one of the highly expressed toxin-like transcripts in *P. clavata* (196 TPM), but it is involved in processing and releasing venom proteins, rather than being a toxin *per se* [17]. Neither of these hits were included in the toxin-like transcript list generated by Tox|Note.

### 2.2. P. clavata Venom Proteome

**Figure 2:**
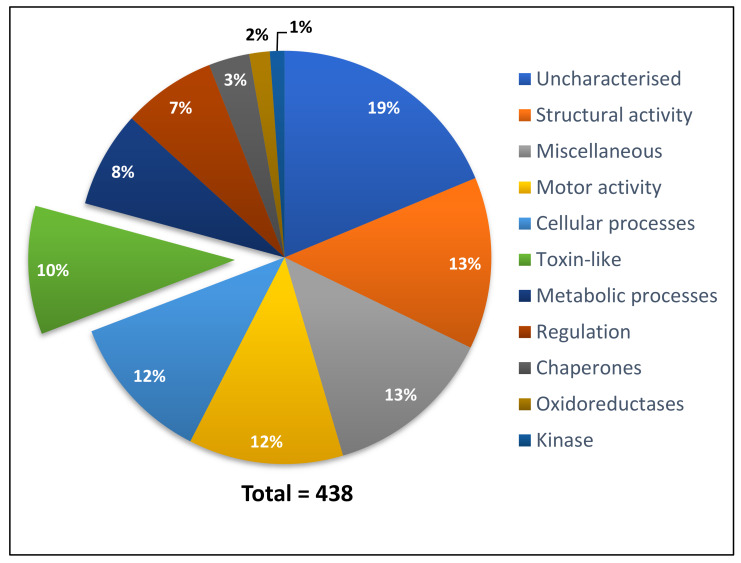
Protein categories detected in *P. clavata* venom. *P. clavata* contigs were identified by mapping the proteome to the transcriptome. Annotation from BLASTx was used to place each contig into one of the above categories. The green portion shows the proportion of toxin-like contigs (10%).

The assembled contigs were translated in six frames using TransDecoder [13], generating 34,586 ORFs. This was then uploaded into PEAKS Studio v8.5 as a database to search the shotgun proteomic LC-MS/MS data. The mass spectra search against the transcriptome assembly was performed using PEAKS Studio v8.5 and generated peptide matches to 438 contigs with a minimum –10lgP score of 15. The hits were manually classified into 11 functional categories based on their BLASTx ID using an e-value cut off of e^-4^ (Figure 2). If a contig had no BLASTx hit, the matched peptides were searched on BLASTp to confirm that the protein was indeed uncharacterised. This approach ensured contigs such as poneratoxin (9279 TPM) were identified, as the BLASTx search identified it as an uncharacterised protein whereas BLASTp confirmed it as poneratoxin. Nevertheless, the majority of proteins detected in the venom were uncharacterised (Figure 2), followed by proteins with structural/ motor or cellular function which is comparable to the GO results from the transcriptome. Among other protein categories were toxin-like proteins, and proteins involved in metabolic processes, and regulation as well as chaperones, oxidoreductases, kinases and miscellaneous proteins. Positive matches between proteins identified by proteomics and transcriptomics allowed for the confirmation of proteomic data as well as complete predicted full-length protein sequences. For a full list of the proteins present in Figure 2, see Supplementary File S2.

The toxin-like category contained a total of 44 contigs, shown in Table 3. The best match, based on a PEAKS score of −10lgP, was to hyaluronidase followed by dipeptidyl peptidase, poneratoxin and phospholipase A_2_. All contigs in Table 3 were also re-named based on BLASTx or BLASTp hits according to the proposed nomenclature systems and queried for signal peptides using SignalP v4.1 (DTU Bioinformatics, Lyngby, Denmark; http://www.cbs.dtu.dk/services/SignalP-4.0/) [18]. Many contigs had no predicted signal peptide despite the fact that they were detected in the venom proteome indicating the presence of non-secreted proteins in our venom sample. These were excluded from our list of toxins.

### 2.3. P. clavata Toxins

Many of the toxin sequences identified in this study have not been previously reported in *P. clavata.* Therefore, we undertook a comparative study with homologous sequences to determine potential pharmacological targets. This was achieved through comparison of critical residues for activity between sets of homologous sequences. Proteins chosen for alignment are detailed below.

#### 2.3.1. Poneratoxin

**Figure 3:**
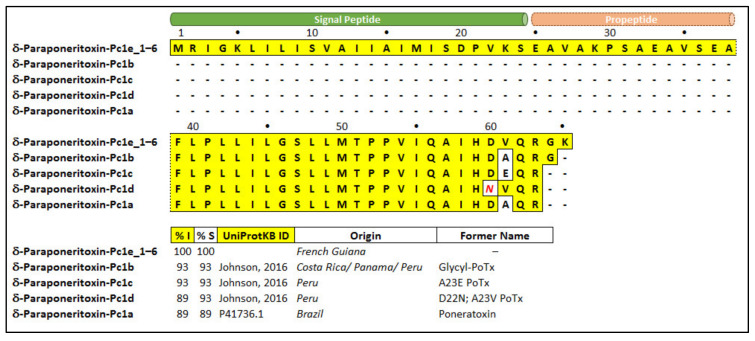
Amino acid sequence alignment of δ-paraponeritoxin-Pc1e isoforms to published δ-paraponeritoxin sequences. Predicted signal peptide (www.cbs.dtu.dk/services/SignalP/) and the predicted propeptide are indicated by green and orange tubes above the sequences, respectively. Identical residues in the peptide sequences are boxed in yellow while conservative substitutions are shown in red italic text. Percentage identity (%I) is relative to δ-paraponeritoxin-Pc1e while percentage similarity (%S) includes conservatively substituted residues. “UniProtKB ID” indicates the UniProt Knowledge base ID code or source reference. Dashes were inserted to maximise alignment. “Origin” refers to the country where the venom was collected. “Former name” refers to the previously published name assigned to that sequence. Previously published sequences (δ-paraponeritoxin-Pc1b, -Pc1c and -Pc1d) represent isoforms of -Pc1a (formerly poneratoxin) [4,10].

We report, for the first time, the protein precursor of poneratoxin, which includes the signal peptide and propeptide. The most abundant toxin identified in the transcriptome of *P. clavata* is poneratoxin, as seen in Table 2, which is consistent with previous investigations [4,5,19]. To determine sequence similarity with the previously identified poneratoxins, the contigs of δ-paraponeritoxin-Pc1e (Pc1e_1-6), from French Guiana (present study), were aligned with previously published poneratoxin sequences from Brazil, Peru, Panama and Costa Rica (Pc1a-d) using Mega 7 software [20]. The six δ-paraponeritoxin-Pc1e isoforms (Pc1e_1x6) have identical amino acid residues, however the nucleotide sequences were different (hence all were labelled ‘Pc1e’). The δ-paraponeritoxin-Pc1e transcripts included a signal peptide (residues 1–24) and a propeptide (25–38) preceding the first amino acid of the published mature ORF (residues 39–65, with a calculated average mass of 2968.7 Da) (Figure 3). 

The mature toxin present in this transcriptome also has an alanine to valine substitution at position 61 (A23V in the mature toxin), and additional GK residues at the C-terminus, compared to the original Brazilian δ-paraponeritoxin-Pc1a [4]. It seems to be most similar to the recently published “glycl-PoTx” [10], re-named here as δ-paraponeritoxin-Pc1b, as it also contains the additional C-terminal glycine but not the lysine residues. Nevertheless, the shotgun proteomics data revealed a mature δ-paraponeritoxin-Pc1e mass of 2783 Da, consistent with the terminal GK residues undergoing cleavage and a loss of 185.1 Da (Figure S2). If these two C-terminal residues are removed from the alignment, δ-paraponeritoxin-Pc1e has 96% identity and 100% similarity to the Peruvian isoform Pc1d (Figure 3).

**Figure 4:**
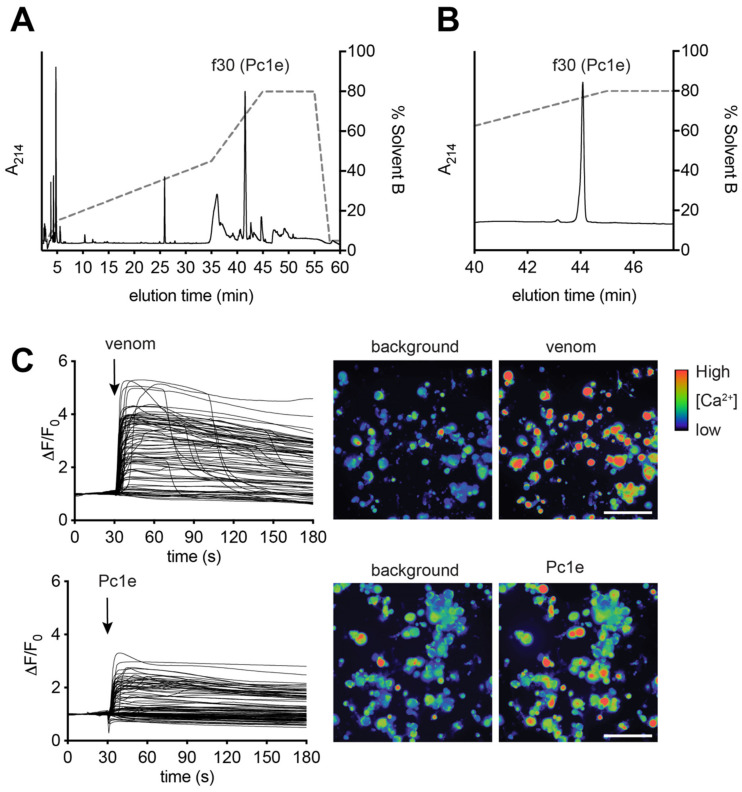
δ-Paraponeritoxin-Pc1e isolation and sensory neuron assay. (**A**) RP-HPLC of *P. clavata* venom revealed a relatively simple profile, with a large late-eluting peak corresponding to δ-paraponeritoxin-Pc1e. (**B**) δ-paraponeritoxin-Pc1e was purified from the venom (Figure S3 illustrates the match to the δ-paraponeritoxin-Pc1e contig). (**C**) Addition of whole *P. clavata* venom (100 µg/mL) to dissociated mouse DRG cells produced an increase in [Ca^2+^]_i_ in all DRG cells, which was particularly pronounced in neuronal cells, and was followed by some dye leakage from a small proportion of cells (see the right hand panels in C representing a snapshot at 180 s). An equivalent amount of isolated δ-paraponeritoxin-Pc1e produced an increase in [Ca^2+^]_i_ specifically in excitable cells. Each trace represents a single DRG cell in the field of view. Snapshots shown of the recording are at 0 s (baseline) and 33 s (3 s after addition of venom or δ-paraponeritoxin-Pc1e). Scale bar: 100 µm.

RP-HPLC of *P. clavata* venom indicated a relatively simple venom composition (Figure 4A), consistent with previous publications [4,5,11]. The RP-HPLC profile is characterised by the majority of peaks eluting at high acetonitrile concentrations, dominated by a large late-eluting peak. Purification of this component (Figure 4B) and sequencing by MS/MS (Figure S3) identified this as poneratoxin (δ-paraponeritoxin-Pc1e). 

We investigated the effects of both the whole venom of *P. clavata* and isolated δ-paraponeritoxin-Pc1e on mammalian sensory neurons using a high-content calcium imaging assay. Application of whole *P. clavata* venom (100 µg/mL) to dissociated mouse DRG cells caused an immediate sharp and sustained (over the 3 min course of the experiment) increase in intracellular calcium concentration ([Ca^2+^]_i_) in all cells (Figure 4C). The largest increases were in neuronal cells, while in a small proportion of cells some dye leakage into the extracellular medium was observed (indicative of mild cytolytic activity). The observed high increase in [Ca^2+^]_i_ observed in DRG cells is consistent with cellular depolarisation and ultimately pain. Isolated δ-paraponeritoxin-Pc1e caused a similar immediate and sustained increase in [Ca^2+^]_i_, but was selective for neuronal cells and was not accompanied by any dye leakage (Figure 4C). These observations show that δ-paraponeritoxin-Pc1e is capable of causing a rapid depolarization of sensory neurons, which is consistent with δ-paraponeritoxin-Pc1e being a major, if not the major, algogenic component in the venom of *P. clavata*. 

#### 2.3.2. Inhibitor Cysteine Knot Peptide

A transcript encoding a peptide highly similar to ant “ω-conotoxin-like” peptide was found in the *P. clavata* venom gland transcriptome (Figure S4). The sequence of the *P. clavata* “ω-conotoxin-like” peptide was 90% identical to that from *Camponatus floridanus*, and 90% homologous to the ω-conotoxin-like protein from ant *Trachymyrmex cornetzi*. Interestingly, identity notably decreased when compared to the same peptide from the bee *Apis florea* (63%). Manual BLASTx results did not produce significant hits to any marine cone snail peptides. We therefore re-searched with the organism filter restricted to “*Conus*” in order to confirm the similarity of these ant “conotoxin-like-proteins” to that of cone snails. As shown in Figure S4, the highest match was to a peptide from *Conus lividus*, and had a very different sequence to all the ant sequences, with an identity of only 15% and similarity of 31% to that of *P. clavata*. The alignment also highlighted that the main conserved amino acids between the sequences were the cysteine residues, which are normally conserved due to their role in the structural framework. Therefore, we did not name the *P. clavata* contig using the same nomenclature as the ant ω-conotoxin-like proteins. Instead, we named it U_1_-paraponeritoxin-Pc1a because the main activity associated with ω-conotoxins—an action to inhibit voltage-gated calcium channels [21]—is very unlikely given the low sequence similarity.

#### 2.3.3. Phospholipase A_2_ (PLA_2_) 

The second most highly expressed toxin transcript in the *P. clavata* venom gland transcriptome after poneratoxin, was a 202-residue phospholipase A_2_ (PLA_2_) (Table 2) (6328 TPM). These proteins have previously been shown to be abundant in *P. clavata* venom [11]. PLA_2_ proteins are not unique to ant venoms, but are found in numerous animal venoms [22,23,24,25]. The size of the *P. clavata* PLA_2_ and the presence of a His48/Asp49 pair suggests that this is a group III PLA_2_ which is also present in other hymenopteran venoms including bees and ants [26,27]. The presence of PLA_2_ in the venom proteome (Table 3) also confirms its secretion in high enough abundance for detection by mass spectrometry. 

**Figure 5:**
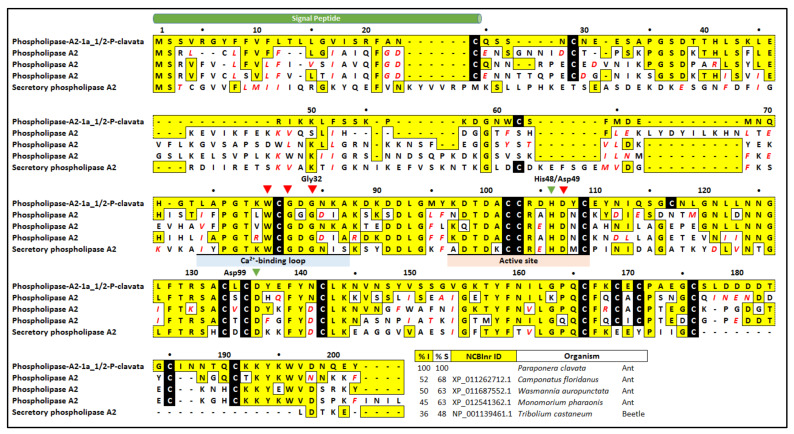
Amino acid alignment of *P. clavata* PLA_2_ isoforms. This figure shows the alignment of *P. clavata* transcripts with PLA_2_-like proteins from the ant species *Camponotus floridanus, Wasmannia auropunctata, Monomorium pharaonis* and the red flour beetle *Tribolium castaneum.* The predicted signal peptide (green tube) is indicated above the sequences. Residues identical to the *P. clavata* peptide sequences are boxed in yellow, while conservative substitutions are shown in red italic text. Cysteines are boxed in black. Gaps were introduced to optimize the alignments. The blue triangle indicates the predicted N-terminus [23]. The underlined coloured regions indicate the Ca^2+^-binding loop (blue), active site (orange) and conserved region (green) [23,26,28]. The red triangles indicate residues critical for calcium binding and the green triangles indicate the active site residues [23,26,29,30]. Percentage identity (%I) is relative to the first peptide of each family, while percentage similarity (%S) includes conservatively substituted residues.

BLASTx analysis identified a number of putatively homologous insect PLA_2_ sequences (Figure 5). The PLA_2_ from *C. floridanus* shared 68% identity while other ant species shared 60% identity. Interestingly, identity was not very high to any of the ant species, with the highest being 52% and the next highest identity to the beetle *Tribolium castaneum* with an identity of 36%. Although the identities were not high, it should be noted that all critical residues were found in the *P. clavata* contig. For example, almost all cysteine residues were present and the previously described calcium binding loop and active site was found across the sequences, particularly the critical His48, Asp49 and Gly32 residues.

#### 2.3.4. Hyaluronidase

A transcript composed of 357 residues with sequence similarity to hyaluronidase was identified by a BLASTx search of the NCBInr database. Its presence and expression in the venom was confirmed through the shotgun proteomics, and showed the highest –10lgP score of 333. There were a total of eight contigs (1a_1 to 1a_8) for hyaluronidase-1a-P-clavata (Figure S5). The translated protein sequence had the highest similarity to a hyaluronidase-like sequence from the ant *Pogonomyrmex barbatus* (85% similarity; Figure S5). All cysteines were conserved across matched proteins with the majority of the differences located near the N-terminus of the sequences. All ant hyaluronidases contain the conserved active site residues previously described in bee venom hyaluronidases (Asp111, Phe112, Glu113, and Glu247; numbered according to the bee venom hyaluronidase) [31].

#### 2.3.5. Icarapin

The protein icarapin was detected in both the proteome and the transcriptome. The transcript icarapin-1-P-clavata is a 163-residue protein that had relatively low similarity to known proteins, with the highest match to icarapin from the ant *Linepithema humile* (58% identity; Figure S6). Interestingly, from residues 73 to 118 (numbering from icarapin-1-P-clavata) all sequences share very high similarity, which is where the consensus icarapin sequence is located [32]. However, the *P. clavata* transcript was not 100% identical to the published sequence [4], as there were a few substitutions (positions 87, 100, 108 and 111) and a threonine insertion at position 92. The role of icarapin in venoms is unclear.

#### 2.3.6. Arginine Kinase

Of all the proteins described here, the most conserved of all matches was that of the 355 residue arginine kinase, with the lowest similarity of 97% (Figure S7). This protein was detected initially by BLASTx homology searching; it was then confirmed through proteomic analysis of the venom. Importantly, all residues critical for arginine binding, ATP binding and catalysis are found amongst all sequences [33]. Additionally, the two specificity loops implicated in the arginine kinase activity and the phosphagen kinase site were also conserved. No signal peptide was predicted by SignalP v4.1 for the *P. clavata* transcript or the other matched proteins. For this reason, we cannot rule out the possibility that this protein serves an endogonous function in the venom gland of *P. clavata*, rather than as a toxin.

#### 2.3.7. Serine Proteases

Serine proteases were the most diverse of the toxin-like transcripts detected in the BLASTx search (Figure S8), although only one serine protease isoform was detected in our venom sample.The longest transcript recovered from our transcriptome translated to 256 amino acid residues and was named serine-protease-5a-P-clavata. Interestingly, we also found transcripts that appear to be truncated versions of isoform 6a, namely isoforms 5a and 8a. These isoforms also had a few non-conserved and conserved substitutions. The BLASTx match with the highest identity was to a serine proteinase stubble protein from the ant *Ooceraea biroi* (81% identity). However, it must be noted that the *O. biroi* protein has 173 amino acid residues preceding the ones shown in Figure S8, which suggests that the *P. clavata* proteins are all truncated versions of this full-length proteinase. An important feature of serine proteinases is the catalytic triad (His37, Asp87 and Ser190, naming according to the *P. clavata* serine proteinase). This was conserved across all the ant serine proteinases (Figure S8) apart from the serine-protease-8a-P-clavata, which has a glycine instead of a serine at position 190. The residues important for the specificity pocket were also present in all of the *P. clavata* isoforms, except serine-protease-5a-P-clavata which had a conservatively substituted alanine instead of a glycine. These proteins may serve an endogenous function e.g., in toxin maturation, in the *P. clavata* venom gland or as toxins themselves.

### 2.4. Novel Toxin-Like Proteins

**Figure 6:**
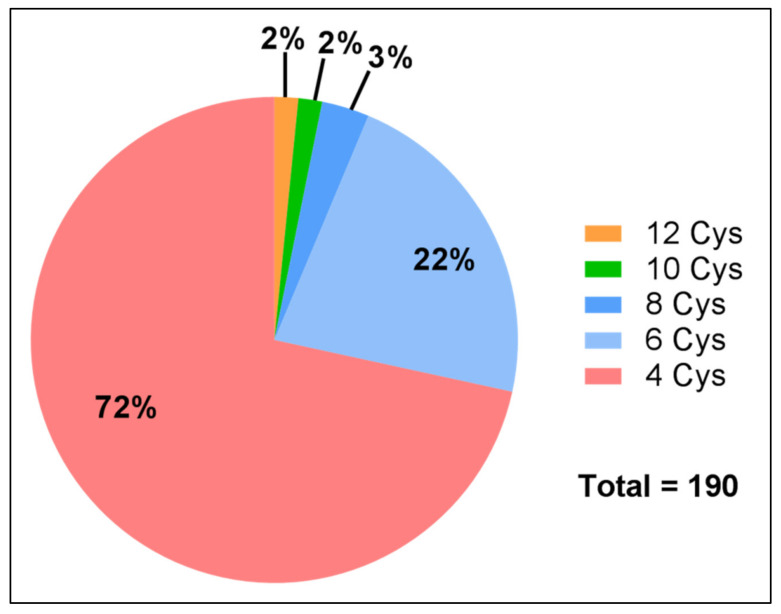
Distribution of novel *P. clavata* toxin-like peptides with four or more cysteines. Predicted peptide toxins were identified by Tox|Note. Upon closer inspection of the sequences that contained six cysteines, seven peptides were found that adhered to a canonical inhibitor cysteine knot (ICK) framework similar to the conotoxin VI/VII framework as shown in Table 4. There were also other peptides conforming to the ICK framework, however, they had eight and 10 cysteines.

In order to identify novel toxin-like peptides in the venom gland transcriptome of *P. clavata*, the transcriptome was analysed with Tox|Note, a pipeline developed mainly for the identification of novel spider-venom toxins [15]. This platform was chosen due to the lack of ant- or hymenopteran-venom specific platforms. The output contains the predicted toxin-like sequences and information regarding the predicted prepropeptides and mature toxin. Predicted peptide toxins were then sorted manually to identify those with a “toxin-like” cysteine framework, defined as peptides with an even number of greater than four cysteines, and then double checked for novelty by BLASTp. As seen in Figure 6, there were a total of 190 peptides conforming to these conditions with the majority (72%) having four cysteines and the highest number of cysteines being 12 which would indicate six disulfide bonds. The disulfide-bond pairs were predicted according to the conotoxin frameworks (www.conoserver.org).

The majority of the peptides with cysteine patterns consistent with an ICK structural framework (containing six or more cysteines conforming to “C-C-CC-C-C”) showed very low expression (Table 4). The highest had a TPM of 44, while conotoxin cysteine framework 1 toxins (containing four cysteines conforming to “CC-C-C”) had higher transcript levels overall, with the highest TPM value of 771 for one of the contigs. Given their low abundance, or possible masking by more highly expressed peptides, we did not find any evidence for these disulfide-rich peptides in the venom proteome. This suggests that while they were annotated by Tox|Note as toxin-like, it is unclear if they represent a large proportion of the venom components in *P. clavata*.

## 3. Discussion

Here, we present the transcriptome and proteome of the bullet ant, *P. clavata*, which is infamous for inflicting the most painful sting among the Hymenoptera [34,35]. Investigating *P. clavata* venom is critical for understanding the basis for its painful venom and also as a source of potential bioactive compounds for development of insecticides or therapeutics [10,36]. We reveal the identification of a novel paraponeritoxin and the first report of the signal and propeptide sequences for this family of toxins. In addition, we have identified the presence of a wide range of toxins that no doubt contribute to the overall toxicity of the whole venom and provide for novel pharmacological lead compounds.

### 3.1. P. clavata Transcriptome

In order to annotate the venom gland transcriptome of *P. clavata*, a BLASTx search of the NCBInr database was performed and generated over 37,000 significant hits (68%) which were queried for GO terms to determine the broad functions of the peptides and proteins. It was found that overall GO terms relating to molecular functions predominated, which was also seen with the *T. bicarinatum* venom gland transcriptome [27] and the sea nettle, *Chrysaora fuscescens*, transcriptome [37]. The most common predicted molecular functions of *P. clavata* venom gland transcripts were binding and catalytic activity and the most common biological processes were related to metabolic and cellular processes, similar to that previously reported for the ant *T. bicarinatum* [27]. Such proteins may be involved in the folding of venom peptides, their cleavage or secretion in the venom gland.

The majority of contigs with BLASTx hits matched transcripts of the ants *H. saltator* and *D. quadriceps* which was not surprising, given the availability of a *D. quadriceps* transcriptome and an *H. saltator* genome [38]. Both of these ants also belong to the subfamily Ponerinae, one of the phylogenetically closest subfamilies to Paraponerinae [39,40]. This is further supported by the lack of matches to *T. bicarinatum,* an ant belonging to the subfamily Myrmicinae, a phylogenetically distant subfamily to Paraponerinae. 

The expression levels of poneratoxin and PLA_2_ (up to 39,657 TPM and 6328 TPM, respectively) was consistent with their detection in the venom here as well as previous 2D-PAGE proteomic studies of *P. clavata* venom [11]. Proteases, which were expressed at relatively lower levels (all <834 TPM), were the most diverse group of toxin-like transcripts, in terms of contig number, with 149 different contigs. Other ant venom gland transcriptomes report allergens and phospholipases as the common proteins [27,36]. The following section summarises the most important characteristics of a selected group of five highly expressed toxin and toxin-like families.

#### 3.1.1. Neurotoxins

*P. clavata* is one of only a few ant species to have had its venom studied extensively and from which a peptide neurotoxin has been sequenced and its pharmacology determined [4,5,10,19]. The pain inflicted by the sting of *P. clavata* has been assumed to derive from poneratoxin (here renamed δ-paraponeritoxin-Pc1a), first identified by Piek [4,5,19]. Surprisingly, our initial BLASTx search of contigs against the NCBInr database did not identify δ-paraponeritoxin-Pc1a as a hit. However, a BLASTp search of peptides identified by LC-MS/MS analysis identified the toxin and matched this sequence to the contigs δ-paraponeritoxin-Pc1e in the translated database generated in this work. One of these contigs (δ-paraponeritoxin-Pc1e_1) displayed very high expression levels (39,657 TPM) and revealed that poneratoxin was indeed the most abundant toxin transcript expressed in the venom gland of *P. clavata*. Importantly, we report, for the first time, a signal peptide and propeptide for a δ-paraponeritoxin. The propeptide comprises the 14-residue segment (EAVAKPSAEAVSEA) following the 24-residue signal peptide (MRIGKLILISVAIIAIMISDPVKS), and the present proteomic experiments as well as previous studies indicate that Phe38 is the N-terminus of the mature toxin [4,5,6,10,19]. This signal peptide seems to be unique to ant venoms, with no sequence similarity reported to date. There appears to be some similarity to the precursor sequence (signal and propeptide) for the aculeatoxins, however this is insufficient to suggest that these sequences arise from an aculeatoxin gene superfamily [41]. Unexpectedly, the Tox|Note BLASTx search did not identify a hit to δ-paraponeritoxin-Pc1a either, which might be due to the algorithm’s generation of incomplete alignments in some cases resulting in an incorrect annotation [15]. Interestingly, Tox|Note then classified the majority of the δ-paraponeritoxin-Pc1e contig as a propeptide, perhaps because the Spider|ProHMM predictive tool used by Tox|Note to predict propeptide cleaving sites was designed to detect spider venom toxins. Most notably, the propeptide in δ-paraponeritoxin-Pc1e seems to be unusual and completely different to the propeptides reported in spiders and cone snails, where a conserved arginine is usually located at position −1 from the N-terminus of the mature peptide [42,43]. Only once there is sufficient transcriptomic and proteomic data for ant venom peptides would it be possible to develop a similar script, capable of predicting propeptides in ants, and perhaps other hymenopterans. 

As seen in Figure 3, the alignment of *P. clavata* transcripts with other homologous δ-paraponeritoxins shows that the isoforms detected as part of this study were similar, but not identical to any of the previously described sequences, differing by only one or two amino acids. This subtle difference between isoforms may cause changes in the activity and specificity of venom peptides. For example, natural mutations in specific regions of δ-paraponeritoxins showed that a decrease in net charge and hydrophobicity at the C-terminus decreased the activity of the toxin while an increase in net charge and hydrophobicity increased the activity [10]. These results highlight the fact that even a subtle change in the primary sequence can result in altered activity, and therefore correct assignment of function can only be determined by activity testing. The additional Gly and Lys residue at the end of the δ-paraponeritoxin-Pc1e transcript is an amidation signal that is cleaved during the maturation of the peptide [44]. However, comparison of the measured mass via MALDI-TOF MS, also collected in French Guiana (2783.4 Da), was consistent with the calculated mass of the *P. clavata* δ-paraponeritoxin-Pc1e transcripts, without the Gly and Lys residues at the C-terminus (2783.6 Da), indicating C-terminal cleavage of these residues and no amidation, or perhaps that these residues are absent in the precursor. 

Although the algesic activity seen following *P. clavata* envenomation has been attributed to δ-paraponeritoxin [4], the present investigation suggests that there are other proteins in this venom that could contribute to the pain-producing activity during envenomation. Nevertheless, the majority of the pain signal portrayed by the sensory neuron assay does implicate δ-paraponeritoxin as the primary agent. 

The majority of the algesic activity observed in the sensory neuron assay suggests δ-paraponeritoxin as the primary pain producing agent, which is consistent with previous studies [4]. Nevertheless, the present holistic proteomic/transcriptomic approach has increased the range of proteins and identified proteins present in ant venom and suggests that there are other proteins in this venom that could contribute to the pain-producing activity during envenomation. For example, the protein arginine kinase has not been previously identified and characterised in ant venoms. Arginine kinases are major components of wasp venoms where they have been associated with the induction of pain and have been shown to be paralytic to spiders in some cases [33,45]. Similar to wasp venoms, *P. clavata* venom seems to have significant levels of this protein as it was found both proteomically and with moderate expression levels in the transcriptome (382 TPM). Importantly, there was a 97% similarity of arginine-kinase-1a-P-clavata to the arginine kinase from the venom of the wasp *Cyphononyx dorsalis,* the first venom in which arginine kinase was identified and found to be paralytic [46]. Additionally, all residues critical for arginine binding, catalysis and ATP binding [33] were conserved in arginine-kinase-1a-P-clavata. It should be noted that although this protein was detected proteomically, no signal peptide was identified, suggesting the ORF is not complete despite the detection of a start (ATG) codon. This suggests that perhaps there is a start codon further upstream or that the protein is not secreted. 

#### 3.1.2. Proteases

Proteases are major components of some venoms where they are involved in digestion [47], haemostasis and thrombosis [48,49] and have also been found to be allergenic [27,50]. They are commonly found in the venoms of wasps [51], bees [47], ants [27] and snakes [48] where they have also been identified to act as factors that facilitate dispersion of other venom components and normal intracellular degradation of proteins [27]. In the present study, proteases were the most common toxin-like transcript in the venom gland transcriptome and isoforms were also detected in the venom. The major proteases were serine proteases, which have been implicated in many biological processes in arthropods such as activation of the Toll signalling cascade and regulation of the polyphenol oxidase activation that affects immune defence mechanisms and inhibition of melanisation [51,52]. Serine proteases found in snake venoms have been implicated in thrombin-like activity, formation of fibrin clots, kininogenase activity and activation of coagulation factor V, platelets or plasminogen [48]. The alignment of the *P. clavata* serine protease sequence with other homologous proteins (see Figure S8) showed that all residues critical for catalytic activity (His37, Asp87, Ser190) corresponding to the “catalytic triad” [47,53] are conserved, except isoform 8, indicating that the majority are likely to retain their catalytic activity. The presence of a glycine instead of a serine residue at position 190 in isoform 8 indicates that this serine protease may belong to the PPAF-II family of serine proteases. PPAF-II serine proteases lack catalytic activity but still have the three disulphide bonds, referred to as a “clip” domain [54], and have been found in solitary wasp venom and more recently in the *D. quadriceps* venom gland transcriptome [36,51]. Interestingly, two truncated versions of this same group of proteins were also identified that may, or may not, be functional. These truncations may also represent an active form of zymogen which has been previously reported in snakes and bees [47].

#### 3.1.3. Phospholipases

One of the enzymes with the highest levels of expression in the *P. clavata* venom gland transcriptome were isoforms of PLA_2_, commonly seen in other ant venom gland transcriptomes [27,36,55]. This is consistent with the large number of spots identified as PLA_2_ proteins in a 2D-PAGE analysis of *P. clavata* venom [11]. Phospholipases are not exclusive to ants, and are found in a wide variety of animal venoms including scorpions [56], snakes [29,57] and bees [23]. Amongst hymenopterans, the transcriptomic data from the ants *T. bicarinatum* and *O. monticola* have been previously reported to have low levels of phospholipase expression, with levels being significantly higher in wasps [27] and the ant *D. quadriceps,* which is consistent with our data [27,36]. 

To date, there are at least 15 classes of secreted PLA_2_s that have been described in the literature including several subtypes within each class [22]. BLASTx searches revealed high similarity of two *P. clavata* transcripts to a PLA_2_ found mainly in hymenopteran venoms (Figure 5). Nevertheless, it can be assumed that this isoform is not myotoxic due to the presence of an aspartate residue instead of a lysine at position 117. *P. clavata* PLA_2_ is also likely to be a group III phospholipase [24] which has been reported in other ant venom transcriptomes [27,36,55]. In support, group III PLA_2_s contain highly conserved active site residues: His48 immediately preceding the calcium binding residue Asp49 as well as a conserved Gly32 [23,25]. 

#### 3.1.4. Hyaluronidases

Hyaluronidases are a class of highly conserved proteins found in almost all venoms and are believed to aid in the distribution and spread of other venom components by hydrolysing hyaluronic acid, a key component of the extracellular matrix of vertebrates [58]. Eight transcripts from the *P. clavata* venom transcriptome had significant similarity to hyaluronidase-like proteins from other ants and their expression was confirmed by the proteomics investigation. Alignment of these sequences with ClustalW confirmed that all residues critical for the tertiary structure (four cysteine residues) and the active site (Asp135, Phe136, Glu137) were conserved [58]. The high sequence conservation across hyaluronidases may play a role in the cross-reactivity seen in allergic reactions to hymenopterans where patients can be allergic to both bee and wasp venoms [58,59]. Ants, however, seem to have less hyaluronidase expression, with the highest level reported from the harvester ant *Pogonomyrmex* from which allergic reactions have been reported [60]. Interestingly, the transcripts with similarity to known hyaluronidases in *P. clavata* were highly expressed*;* therefore, they may induce similar activity in their victims. However, a biochemical characterisation is necessary to understand its true role as no allergic reactions have been reported following *P. clavata* envenomation [34].

#### 3.1.5. Allergenic Proteins

There were also hits to proteins such as icarapin and pilosulin 4, both of which are allergenic proteins found in ant venoms. Icarapin is a protein first isolated from the honeybee, *A. mellifera,* and is involved in IgE binding [32]. Recently, icarapin was reported in the transcriptome of the ant *O. monticola*, which is the first report of this peptide in ant venoms. The high similarity between the transcripts identified in this transcriptome with that of the bee *A. mellifera* suggests that it might have a similar role in this venom. Pilosulin 4, an ant venom allergenic peptide originally isolated from the ant *Myrmecia pilosula*, was also identified in the *P. clavata* venom gland transcriptome (Supplementary File S1). However, the low TPM value of this transcript (8 TPM) suggests that this peptide may not play a major role in the venom of *P. clavata.*

### 3.2. Protein and Peptide Transcripts Not Detected in the Venom Proteome

#### 3.2.1. Toxin-Like Proteins

Several transcripts not detected in the venom proteome displayed similarity to toxins without a known activity such as U_8_-agatoxin-Ao1a-like protein from the spider *Agelena orientalis* and a number of “ω-conotoxin-like” peptides from other ants. These peptides are yet to be investigated for their pharmacological activity. However, alignment of these other ant peptides, and those from *P. clavata* (Figure S4) with an actual ω-conotoxin peptide from the marine cone snail C*onus lividus* revealed that there was very little similarity to that of the cone snail aside from the cysteine framework (15% identity). ω-Conotoxins are known to block voltage-gated calcium channels and result in analgesic effects [21,61]. However, the activity of the *P. clavata* transcript will need to be tested in order to identify its target, particularly because *P. clavata* is known for its algesic, not analgesic, effects [34,35]. Therefore, the transcripts isolated from *P. clavata* have been named U₁-paraponeritoxin-Pc1a, based on the King (2008) nomenclature systems for toxins with unknown activity [62].

#### 3.2.2. Insecticidal Proteins 

Three transcripts with similarity to sphingomyelinase phosphodiesterase proteins (highest: 27 TPM) were identified in this investigation but were not found in the venom proteome by MS analysis. Sphingomyelinase D is a spider venom toxin that has been identified as a potent dermonecrotic protein as well as an insecticidal protein, as it has a role in immobilising prey [63]. This is highly interesting as this is the first report of this protein in a venom gland transcriptome other than that of the *Loxosceles* spider [64]. This finding suggests that the insecticidal activity seen in *P. clavata* might not be due to poneratoxin alone, as previously suggested [8], but rather a combination of several insecticidal components. However, the sting from *P. clavata* has never been reported to cause dermonecrosis.

#### 3.2.3. Other Predicted *P. clavata* Toxin-Like Peptides

An interesting finding of the present study was the discovery of several peptides that have the potential of being novel bioactive molecules. Tox|Note identified hundreds of transcripts as encoding novel predicted toxins. Cysteine-rich peptides were of particular interest due to their high diversity, stability and likelihood of bioactivity [65]. Further analysis of these sequences revealed several peptides conforming to the inhibitor cystine knot (ICK) framework. This is a highly stable protein fold with a characteristic “pseudo-knot” formed by an α-helix, β-sheet or random coil connected by two disulphide bonds and interlaced with a third disulphide bond [66]. ICK peptides are yet to be identified in ant venoms, only recently identified in the venom gland transcriptome of *T. bicarinatum.* That study also reported only a single peptide conforming to this framework and with only low expression levels [36]. The present study identified six peptides with cysteines conforming to the general ICK framework. However, these had low expression levels, with TPM values ranging from 3 to 44, and were not detected in the venom proteome. 

One of the highly expressed toxin-like peptides identified by Tox|Note (771 TPM) was a peptide conforming to conotoxin cysteine framework I, also observed with α-conotoxins [67]. We found nine transcripts with this framework which is the first report of this type of peptide in ant venom transcriptomes. It would be interesting to test the activity of these toxins, as α-conotoxins are known for their activity on nicotinic acetylcholine receptors [61]. Medical applications of these peptides include potential use in neuropsychiatric disorders such as Parkinson’s and Alzheimer’s as this receptor has been implicated in their pathophysiology [67].

### 3.3. Combined Proteomic/ Transcriptomic Approach

The identification of individual peptides from complex mixtures such as venom remains a challenge despite recent advances in technologies. In fact, there is no single method capable of identifying all components of a given sample. For example, RNAseq alone would not have detected poneratoxin, which was identified through proteomic approaches. Several groups are therefore now moving to a combined proteomic / transcriptomic methodology in an attempt to overcome this issue [37,55]. Unsurprisingly, minimal overlap has often been obtained between the two techniques [37]. This was also the case in our investigation, as only 44 of the predicted 453 toxin-like transcripts identified in the transcriptome were identified in the proteome. However, this may be a taxa-dependent issue or a technical issue as approximately 90% overlap was obtained between the peptidome and transcriptome of the cone snail *Conus marmoreus* when using triple quadrupole mass spectrometry and high-depth pyrosequencing [68]. Therefore, in the future, high resolution mass spectrometry coupled to high resolution chromatography should be employed to try and maximise this overlap. The low overlap might be due to the inherent limitations in resolution of either technique. Furthermore, the fact that venoms are a mixture of hundreds of components often with similar molecular size, isoelectric point, and closely related isoforms with different expression levels, all makes HPLC separation difficult. The poor overlap may also be due to the many potential PTMs in the peptides/proteins.

There are many advantages to this holistic approach, as it allows for confirmation of translation of certain transcripts found within the transcriptome. It also allows for better protein identification by proteomics with a bottom-up approach [3]. This is exemplified in the present study where four times the number of proteins were detected compared to our previously published *P. clavata* proteome [11]. In the previous study we found a total of 95 proteins, whereas here we report 495 using the shotgun approach. There are many reasons for this discrepancy, one is no doubt the improved sensitivity of the mass spectrometer employed in the present investigation. It is also noteworthy that according to the transcriptome, there should be over 54,000 expressed proteins with differing expression levels. However, a transcriptome is representative of transcripts being transcribed at the point of time that they were collected, and one cannot assume that all are expressed at the protein level. Nevertheless, there is no clear explanation as to why the correlation remains so imperfect, and further work needs to be undertaken in order to improve the match between venom proteome and transcriptome.

## 4. Conclusions

This study presents the first holistic investigation of the venom of the bullet ant *P. clavata*, with a focus on the toxin proteins and peptides which could be responsible for the extreme pain elicited during envenomation. Venom gland transcriptomics and proteomics resulted in an in-depth coverage of the venom profile of *P. clavata* and revealed a number of highly expressed toxins including neurotoxins, proteases, PLAs and hyaluronidase that likely contribute to the overall toxicity of the venom. We confirmed δ-paraponeritoxin (formerly poneratoxin) as the major toxin in this venom that produces significant activation of primary afferent neurons along with the presence of high levels of PLA_2_. Additionally, δ-paraponeritoxin-Pc1e is a novel paralog not previously identified in the venoms of *P. clavata* from Peru, Brazil, Panama or Costa Rica. 

## 5. Materials and Methods 

### 5.1. Venom Gland Transcriptomics

*Paraponera clavata* worker ants were collected in the locality of “la Montagne de Kaw” in French Guiana (N4° 38′25″ W52° 17′33″). Both venom glands and venom sacs of 52 worker ants were dissected in distilled water and immediately placed into 1 mL of RNAlater [69]. The samples were stored at −80 °C prior to RNA extraction. The RNAlater was removed with a glass Pasteur pipette and the glands disrupted with a TissueLyser II (Qiagen, Germantown, MD, USA) in RLT buffer containing 10% (*v*/*v*) of 2-mercaptoethanol (Rneasy Mini Kit, Qiagen). RNA was first isolated with a phenol-chloroform (5:1) solution followed by washing with a solution of chloroform-isoamyl alcohol (25:1) to remove any traces of phenol. The RNA was then bound to a Qiagen column and washed as per the manufacturer’s instructions. DNAse I (Roche Diagnostics GmbH, Mannheim, Germany) was added in order to remove any remaining fragments of DNA. The RNA was eluted in sterile water and total RNA was determined using a Qubit 3.0 fluorometer (Thermo Fisher Scientific, Waltham. MA, USA) with the RNA HS assay kit (Life Technologies Corp., Carlsbad, CA, USA). A NanoDrop 2000 UV-Vis spectrophotometer (Thermo Fisher Scientific) was used to determine 260/280 and 260/230 nm ratios. Finally, RNAsTable™ LD (Biomatrica, San Diego, CA, USA) was added to the purified RNA and the sample dried using a Speed Vac (RC1010, Jouan, Saint Herblain, France). 

Total RNA was dried with RNAstable^®^ reagent (Biomatrica, San Diego, CA, USA) and shipped to the Department of Biological Sciences, National University of Singapore for transcriptomics analysis. The dried sample was resuspended in 41 µL Molecular Biology Grade water and RNA quality and quantity was further assessed using Agilent 2100 Bioanalyzer. Using approximately 850 ng of total RNA, bead-based Poly-AAA tail selection was performed to purify mRNA. A cDNA library was constructed using the NEBNext Ultra Directional Library Prep Kit according to the manufacturer’s protocol. Fragment size distribution of the library was verified using an Agilent 2100 Bioanalyzer. The 250 bp paired-end library was sequenced on an Illumina HiSeq 2500 sequencer on 1/14th of a lane. Due to the small amount of venom gland material from ants, no replicates were made. A summary of the combined proteomic and transcriptomic investigation of the bullet ant *P. clavata* venom is shown in Figure S9.

### 5.2. Quality Control and de novo Transcriptome Assembly

Quality filtering of sequences was performed on the raw Fastq files for using FastQC version 0.115 [70], and fastq-screen (version 0.9.3, Babraham Bioinformatics, Cambridge, UK) (https://www.bioinformatics.babraham.ac.uk/projects/fastq_screen/). Primer and adaptor sequences and low quality bases were trimmed using Trimmomatic (version 0.36, KBase module; https://narrative.kbase.us/#catalog/modules/kb_trimmomatic) with default settings [71]. The resulting paired-end reads were assembled *de novo* using Trinity (version 2.2.0, Broad Institute Cambridge, MA, USA; https://github.com/trinityrnaseq/trinityrnaseq/releases) with default parameters that included in silico normalisation [13]. Bowtie was used to assess the assembly quality by mapping the reads to the contigs [72]. Expression levels were computed in transcripts per million (TPM) using the RSEM package [73]. 

### 5.3. Venom Gland Transcriptome Analysis

#### 5.3.1. BLASTx

In order to annotate the *P. clavata* venom gland transcriptome, all assembled contigs were queried against the NCBI non-redundant online database (National Center for Biotechnology Information, August 2017) using a BLASTx algorithm. All sequences with hits to the database with an e-value below 1e^−4^ were considered for further analysis. Translated protein sequences were assigned Gene Ontology (GO) terms using UniProtKB (Figure S10 [74,75]. Toxins were identified by a manual search of ca. 50 keywords, including toxin, phospholipase, metalloproteinase, acid phosphatase and dipeptidyl peptidase (See Table S1 for a list of all terms). The top 20 expressed toxin and toxin-like transcripts, based on TPM value, were annotated based on their matched proteins and re-named according to the nomenclature system in Section 5.3.6.

#### 5.3.2. TransDecoder

The assembled and annotated venom gland transcriptome was translated using TransDecoder (version 3.0.1) [13,76] using the parameters for six-frame translation with a minimum ORF of 50 amino acids. The resulting ORFs were uploaded onto PEAKS 8.5 as a database (Bioinformatics Solutions, ON, Canada) [77]. This transcriptome database was used to query the LC-MS/MS data obtained from the shotgun mass spectrometry experiment. The peptides from LC-MS/MS which matched to contigs from the translated transcriptome were also searched on BLASTp to confirm the BLASTx match assigned to the contig [14]. 

#### 5.3.3. Signal Peptide Prediction

The signal peptide cleavage site was predicted using the SignalP server (http://www.cbs.dtu.dk/services/SignalP/) [18]. Propeptides could not be predicted, as there is no available predictor for ant venom peptides. These were therefore determined only if proteomic data was available for that transcript.

#### 5.3.4. Prediction of Toxin-Like Peptides

To predict novel toxin-like peptides, the assembled transcriptome fasta file was uploaded onto the Arachnoserver’s Tox|Note pipeline [15] since a similar pipeline for ants, or hymenopterans in general, is unavailable. The pipeline annotates transcripts by similarity-based putative homology or *de novo* using BLASTx or the Tox_Seek| tool, respectively. The Tox|Note output also predicts the transcript cleavage sites for the signal peptide and a propeptide using a combination of the SignalP and Spider|ProHMM tools, respectively [15]. A toxin was considered novel if it had no BLASTx or Tox|Note match. 

#### 5.3.5. Toxin Identification

Toxin matches from BLASTx and Tox|Note were compiled and sorted based on their respective expression TPMs. A manual curation of these proteins was performed by checking the NCBI BLASTx results and through a ClustalW multiple alignment on homologous proteins with Mega7 [20] 

#### 5.3.6. Nomenclature

Throughout this paper, peptide toxins (<10 kDa) have been named according to the proposed rational nomenclature system for peptide toxins from animal venoms of King et al. [62]. This same nomenclature was used to re-name known ant venom peptide toxins in Touchard et al. [78]. A brief summary of the naming system is provided in Figure S11. However, as this rational nomenclature system did not consider proteins, we adapted it to include putative protein toxins. The proposed protein toxin nomenclature method begins with the generic protein name (if known), which is based on a previously assigned name based on homology to other proteins of the same sequence and activity, for example hyaluronidase or phospholipase. If the protein has not been characterised, then the prefix is designated as “Uncharacterised” (U). This generic toxin name is followed by a number that designates a particular family of paralogous toxins with the same activity assigned to the generic protein name. This designator was introduced because often there is more than one group of proteins from the same species that act on the same molecular target. This designator is simply incremented as new groups of toxins are discovered. The toxin-family designator is followed by a lowercase letter that is used to distinguish isoforms as many venomous animals diversify their toxin repertoire through post-translational modifications that alter the original toxin by a few amino acids. However, if the isoform from a transcript is identical at the protein level with a different nucleotide sequence, a numeral is added after the isoform letter preceded by an underscore. The last part of the protein name is an uppercase letter that identifies the genus of origin and the lowercase species name. Both genus and full species name are required to avoid confusion because of the large number of ant species. An example of a protein isolated from the present investigation is provided in Figure S12. 

#### 5.3.7. Identification of Potential Pharmacological Targets

In order to assess the extent of similarity of contigs to the matched proteins and to assess whether the critical residues were present, several potential toxin contigs were manually searched using BLASTx. These were then aligned using the MEGA’s ClustalW alignment feature [20].

#### 5.3.8. Transcriptome Data Submission

All annotated transcripts and sequence data are being deposited into the EMBL-EBI European Nucleotide Archive (ENA).

### 5.4. Venom Collection for Proteomics and Neuron Cell Assay

After collection, ants were stored at −20 °C prior to manual dissection of the venom reservoirs. After dissection, the reservoirs were pooled in 10% (*v*/*v*) acetonitrile (ACN)/water. Samples were then centrifuged for 5 min at 14,400 rpm (12,000 *g*_av_) and the supernatant was collected and lyophilised prior to storage at −20 °C.

To identify proteins present in the venom reservoir, we used a bottom-up proteomics approach. Crude venom was re-suspended in MilliQ water and digested using trypsin (Promega, Madison, WI, USA). Peptides were then analysed using an Eksigent 415 autosampler connected to a 415 nanoLC system (Eksigent, Dublin, CA, USA). Five µL of the sample was loaded at 300 nl/min with MS buffer A consisting of 2% ACN + 0.2% formic acid (FA) by direct injection onto a PicoFrit column (75 mm × 150 mM; New Objective, USA) packed with C18AQ resin (1.9 µm, 200 Å, Dr. Maisch, Germany). Peptides were eluted from the column and into the source of a 6600 TripleTOF hybrid quadrupole-time-of-flight mass spectrometer (Sciex, Redwood City, CA, USA) using the following gradient: 2%–35% MS buffer B (80% ACN + 0.2% FA) over 90 min, 35%–95% MS buffer B over 9 min, 95% MS buffer B for 9 min, 95%–2% for 2 min. The eluting peptides were ionised at 2300 V. An Intelligent Data Acquisition (IDA) experiment was performed, with a mass range of 350–1500 Da continuously scanned for peptides of charge state 2^+^–5^+^ with an intensity of more than 400 counts/s. Up to 50 candidate peptide ions per cycle were selected and fragmented and the product ion fragment masses measured over a mass range of 100–2000 Da. The mass of the precursor peptide was then excluded for 15 seconds. 

### 5.5. Protein Identification

Proteins present in the venom were identified by mapping the mass spectra to the translated transcriptome assembly combined with a contaminants database using the software PEAKS Studio v8.5 (BSI, Waterloo, ON, Canada). Since there is a known variability of toxins post-translationally, several parameters were employed in order to maximise the identified proteins. These parameters included a semi-tryptic peptide with biological modification of deamidation and oxidation and a parent mass error tolerance of 50 ppm. They also included a fragment mass error tolerance of 0.1 Da and use of the enzyme trypsin, with a maximum number of three missed cleavages. The results of the search were then filtered to include peptides with a –log10P score that was determined by the false discovery rate (FDR) of <2%, the score being that where decoy database search matches were <2% of the total matches to call a positive hit. Sequences with less than 95% confidence were excluded. At least two peptides from the shotgun MS/MS analysis were required for protein identification using PEAKS.

### 5.6. Purification of δ-paraponeritoxin-Pc1e by RP-HPLC 

Whole venom (0.5 mg) was separated on a Phenomenex Gemini NX-C18 column (250 × 4.6 mm, 3 µm particle size, 110 Å pore size) using a gradient of 15%–45% solvent B (90% ACN, 0.05% TFA) over 30 min at a flowrate of 1 mL/min. Fractions were collected based on absorbance at 214 nm, and the identity of the major peak confirmed by mass spectrometry as δ-paraponeritoxin-Pc1e. An aliquot of the purified fraction was rerun under the same conditions to assess purity. Purified δ-paraponeritoxin-Pc1e was dried by vacuum concentration and resuspended in 100 µL pure water from which a 6 µL aliquot was used for LC-MS/MS analysis, and 7 µL aliquot was used for calcium imaging experiments. 

### 5.7. LC-MS/MS 

An aliquot of purified δ-paraponeritoxin-Pc1e was reconstituted in 0.5% FA, and separated on a Shimadzu (Tokyo, Japan) Nexera uHPLC with an Agilent Zorbax sTable-bond C_18_ column (2.1 × 100 mm, 1.8 μm particle size, 300 Å pore size). A flow rate of 180 μL/min was used with a gradient of 1%–40% solvent B (90% ACN, 0.1% FA) in 0.1% FA over 45 min and analysed on an AB Sciex 5600 TripleTOF mass spectrometer equipped with a Turbo-V source heated to 550 °C. MS1 survey scans were acquired at 300–1800 *m*/*z* over 250 ms, and the 20 most intense ions with a charge of +2 to +5 and an intensity of at least 120 counts were selected for MS2. The unit mass precursor ion inclusion window mass ± 0.7 Da, and isotopes within ±2 Da were excluded from MS2, with scans acquired at 80–1400 *m*/*z* over 100 ms and optimized for high resolution.

### 5.8. Calcium Imaging of Sensory Neurons

Dorsal root ganglia (DRG) from a 50-day-old C57BL/6 mouse were dissociated, plated in DMEM (Gibco, MD, USA), 10% (*v/v*) foetal bovine serum (Assaymatrix, Melbourne, VIC, Australia), 1× penicillin/streptomycin (Gibco, Gaithersburg, MD, USA) on a 96-well poly-D-lysine-coated culture plate (Corning, ME, USA) and maintained overnight. Cells were loaded with Fluo-4 AM calcium indicator, according to the manufacturer’s instructions (Thermo Fisher Scientific). After loading (1 h), the dye-containing solution was replaced with an assay solution (1X Hanks’ balanced salt solution, 20 mM HEPES). Fluorescence corresponding to intracellular calcium concentration ([Ca^2+^]*_i_*) of 100–150 DRG cells were monitored in parallel using a Nikon Ti-E Deconvolution inverted microscope, equipped with a Lumencor Spectra LED Lightsource. Images were acquired at 20× objective at 1 fps (excitation 485 nm, emission 521 nm). Baseline fluorescence was monitored for 20 s, and at 30 s the assay solution was replaced with an assay solution containing whole venom (100 µg/mL) or purified δ-paraponeritoxin-Pc1e (1:10 dilution of resuspended fraction as described above). Experiments involving the use of mouse tissue were approved by the University of Queensland animal ethics committee, approval code: TRI/IMB/093/17, approval date: 31 March 2017. 

## Figures and Tables

**Table 1:** Assembly statistics and downstream metrics from the *P. clavata* venom gland transcriptome analysis.

After QC	Counts
Total number of reads (paired end)	17,026,962
**Assembly summary**	
Total number of contigs	54,242
GC content	37.3%
Raw reads mapped to contigs	99.3%
**Annotation**	
Total BLASTx hits	37,140
Assembled contigs with BLASTx hits	32, 003
Contigs with gene ontology annotation	6732
At least one ORF	31,586
* Toxins	295
Proportion of contigs that are toxins	4.4%

* Only toxins with a TPM ≥ 1 are included.

**Table 2:** Top 20 most expressed (based on TPM) toxin and toxin-like transcripts from the *P. clavata* venom gland transcriptome BLASTx and Tox|Note searches. A complete list of all the BLASTx toxin and toxin-like transcripts can be found in Supplementary File S1.

Toxin Name	BLASTx/ Tox|Note Protein Name	NCBInr Accession	Species	Trinity ID	TPM	Toxin Category
δ-Paraponeritoxin-Pc1e_1	Poneratoxin	P41736.1	*Paraponera clavata*	DN10254_c0_g26_i11	39,657	Neurotoxic
δ-Paraponeritoxin-Pc1e_2				DN9710_c0_g11_i2	9279	
δ-Paraponeritoxin-Pc1e_3				DN10254_c0_g26_i12	2414	
δ-Paraponeritoxin-Pc1e_4				DN10254_c0_g26_i5	797	
Phospholipase-A2-1a_1-P-clavata	PREDICTED: phospholipase A2	XP_011262712	*Camponotus floridanus*	DN9894_c0_g8_i1	6328	Phospholipase
Translationally-controlled-tumor-protein-1a-P-clavata	PREDICTED: translationally controlled tumour protein homolog	XP_014489127	*Dinoponera quadriceps*	DN9555_c0_g10_i3	1229	Inflammation
Icarapin-1a-P-clavata	PREDICTED: icarapin-like	XP_011863857	*Vollenhovia emeryi*	DN9969_c1_g1_i4	969	Allergenic
Serine-protease-1a-P-clavata	Serine proteinase stubble	EZA53191.1	*Ooceraea biroi*	DN10218_c2_g11_i1	835	Protease
Serine-protease-2a-P-clavata				DN10218_c2_g7_i1	364	
Arginine-kinase-1a_1-P-clavata	PREDICTED: arginine kinase isoform X1	XP_014467324	*Dinoponera quadriceps*	DN9942_c0_g1_i6	382	Neurotoxic
U_1_-Paraponeritoxin-Pc1a	PREDICTED: omega-conotoxin-like protein 1	XP_011257508	*Camponotus floridanus*	DN5808_c0_g1_i1	253	Unknown
Serine-protease-3a-P-clavata	PREDICTED: serine protease easter-like isoform X2	XP_014488937	*Dinoponera quadriceps*	DN10218_c1_g1_i2	161	Protease
Serine-protease-4a-P-clavata	Serine protease snake	KMR05322.1	*Lasius niger*	DN11547_c0_g1_i1	138	Protease
Venom-allergen3-1a-P-clavata	PREDICTED: venom allergen 3-like	XP_012534360	*Monomorium pharaonis*	DN10202_c0_g4_i2	127	Allergenic
Cysteine-rich protein-1a-P-clavata	Cysteine-rich protein 1	KYN02327.1	*Cyphomyrmex costatus*	DN7413_c0_g4_i1	123	Ion channel modulator
Serine-protease-1b-P-clavata	PREDICTED: serine protease 42-like isoform X3	XP_014488938	*Dinoponera quadriceps*	DN10218_c2_g8_i1	113	Protease
Serine-protease-5a-P-clavata				DN10218_c2_g6_i7	103	
Disintegrin-and-metalloproteinase-domain-containing-protein-1a-P-clavata	Disintegrin and metalloproteinase domain-containing protein	XP_012242558	*Bombus impatiens*	DN21256_c0_g1_i1	113	Metalloproteinase
Disintegrin-and-metalloproteinase-domain-containing-protein-2a-P-clavata		EZA52689.1	*Ooceraea biroi*	DN20154_c0_g1_i1	75	
Lysosomal-aspartic-protease-1a-P-clavata	PREDICTED: lysosomal aspartic protease	XP_014468660	*Dinoponera quadriceps*	DN7128_c0_g1_i1	111	Protease
Defensin-2-like-1a-P-clavata	PREDICTED: defensin-2-like	XP_012537462	*Monomorium pharaonis*	DN5917_c0_g1_i1	89	Antibacterial
Serine-protease-inhibitor-1a-P-clavata	PREDICTED: serine protease inhibitor 3/4-like isoform X2	XP_012539061	*Monomorium pharaonis*	DN9965_c0_g10_i1	86	Protease
Serine-protease-6a-P-clavata	PREDICTED: venom serine protease-like	XP_014477136	*Dinoponera quadriceps*	DN9366_c0_g3_i1	85	Protease
Serine-protease-7a-P-clavata	PREDICTED: serine protease 52-like	XP_011641802	*Pogonomyrmex barbatus*	DN10218_c2_g11_i2	84	Protease
Serine-protease-8a-P-clavata	PREDICTED: serine protease snake-like	XP_011142823	*Harpegnathos saltator*	DN9987_c0_g2_i9	83	Protease
Cathepsin-L-1a-P-clavata	Cathepsin L	EFN75465.1	*Harpegnathos saltator*	DN9254_c0_g3_i1	77	Protease

**Table 3:** *Paraponera clavata* toxins identified by proteomic analysis and their BLASTx hit. The table shows transcripts which had at least two peptides from the shotgun MS/MS analysis identified using PEAKS. Also included are the BLASTx match (or BlastP if no match by BLASTx) for each transcript. Additional information regarding transcripts, accessions and full protein names can be found in Supplementary File S2. The presence (Y), or absence (N), of a predicted signal peptide is denoted in the last column of the table

Toxin Name	BLASTx Hit	Species	Trinity ID	Unique Peptide	% Cover	−10lgP	TPM	SigP (y/n)
Hyalyronidase-1a_1-P-clavata	PREDICTED: hyaluronidase-like	*D. quadriceps*	DN10062_c0_g13_i9	70	65	333	38	Y
Hyalyronidase-1a_2-P-clavata			DN10062_c0_g13_i12	70	65	333	23	Y
Hyalyronidase-1a_3-P-clavata			DN10062_c0_g13_i7	70	65	333	3	Y
Hyalyronidase-1a_4-P-clavata			DN10062_c0_g13_i16	70	65	333	3	Y
Hyalyronidase-1a_5-P-clavata			DN10062_c0_g13_i15	70	65	333	1	Y
Hyalyronidase-1a_6-P-clavata			DN10062_c0_g13_i3	70	65	333	1	Y
Hyalyronidase-1a_7-P-clavata			DN10062_c0_g13_i10	70	65	333	1	Y
Hyalyronidase-1a_8-P-clavata			DN10062_c0_g13_i5	70	65	333	1	Y
Venom-dipeptidyl-peptidase-1a_1-P-clavata	PREDICTED: venom dipeptidyl peptidase 4	*L. humile*	DN8858_c0_g1_i1	26	29	281	60	N
Venom-dipeptidyl-peptidase-1a_2-P-clavata	PREDICTED: venom dipeptidyl peptidase 4		DN8858_c0_g1_i2	26	29	281	21	N
δ-Paraponeritoxin-Pc1e_2	Poneratoxin	*P. clavata*	DN9710_c0_g11_i2	20	45	260	9279	Y
δ-Paraponeritoxin-Pc1e_5			DN10254_c0_g26_i8	20	45	260	2	Y
δ-Paraponeritoxin-Pc1e_6			DN10254_c0_g26_i3	20	45	260	0	Y
Phospholipase-A2-1a_1-P-clavata	PREDICTED: phospholipase A2	*C. floridanus*	DN9894_c0_g8_i1	24	48	235	6328	Y
Phospholipase-A2-1a_2-P-clavata			DN9894_c0_g2_i2	24	48	235	7	Y
Venom-allergen3-1a-P-clavata	PREDICTED: venom allergen 3-like	*M. pharaonis*	DN10202_c0_g4_i2	14	44	198	127	Y
Venom-allergen3-1b-P-clavata			DN10202_c0_g4_i1	14	53	198	1	Y
Carboxypeptidase-Q-1a_1-P-clavata	PREDICTED: carboxypeptidase Q	*C. biroi*	DN8288_c0_g1_i2	12	22	180	3	N
Carboxypeptidase-Q-1a_2-P-clavata			DN8288_c0_g1_i1	12	23	180	2	N
Arginine-kinase-1a_1-P-clavata	PREDICTED: arginine kinase isoform X1	*D. quadriceps*	DN9942_c0_g1_i6	10	32	169	382	Y
Arginine-kinase-1a_2-P-clavata			DN9942_c0_g1_i1	10	32	169	4	N
Pancreatic-lipase-1a-P-clavata	Pancreatic lipase-related protein 2	*C. floridanus*	DN10238_c1_g31_i1	8	23	169	15	Y
Pancreatic-lipase-1b-P-clavata			DN10238_c1_g10_i1	8	25	169	3	Y
Serine-protease-6a-P-clavata	PREDICTED: venom serine protease-like	*D. quadriceps*	DN9366_c0_g3_i1	6	10	135	85	Y
Matrix-metalloproteinase-14-like-1a-P-clavata	Matrix metalloproteinase-14	*C. biroi*	DN18586_c0_g1_i1	3	23	104	5	N
Serine-protease-inhibitor-1a-P-clavata	PREDICTED: serine protease inhibitor 3/4-like isoform X2	*M. pharaonis*	DN9965_c0_g10_i1	4	11	97	86	Y
Alaserpin-X5-like-1a-P-clavata	PREDICTED: alaserpin-like isoform X5	*P. barbatus*	DN9965_c0_g10_i2	4	12	97	18	Y
Pancreatic-triacylglycerol-lipase-X1-like-1a-P-clavata	PREDICTED: pancreatic triacylglycerol lipase-like isoform X1	*D. quadriceps*	DN8997_c0_g2_i2	3	6	93	5	Y
Pancreatic-triacylglycerol-lipase-X1-like-2a-P-clavata			DN8997_c0_g2_i3	1	3	37	2	Y
Pancreatic-triacylglycerol-lipase-X1-like-3a-P-clavata			DN8997_c0_g2_i1	1	8	37	1	Y
Venom-acid-phosphatase-1a-P-clavata	PREDICTED: venom acid phosphatase Acph-1	*C. biroi*	DN5022_c0_g1_i1	3	7	63	5	N
Iron-zinc-purple-acid phosphatase-like-1a_1-P-clavata	Iron zinc purple acid phosphatase-like protein	*L. niger*	DN8672_c0_g1_i2	2	4	55	17	N
Iron-zinc-purple-acid phosphatase-like-1a_2-P-clavata			DN8672_c0_g1_i1	2	4	55	9	N
Alkaline-phosphatase-1a_1-P-clavata	PREDICTED: alkaline phosphatase 4-like	*L. humile*	DN9670_c0_g1_i1	1	2	51	24	N
Alkaline-phosphatase-1a_2-P-clavata	Alkaline phosphatase 4	*H. saltator*	DN9670_c0_g1_i2	1	2	51	2.3	N
Neprilysin-11-isoform-X2-1a_1-P-clavata	PREDICTED: neprilysin-11 isoform X2	*D. quadriceps*	DN10125_c0_g9_i1	2	4	43	4.5	Y
Neprilysin-11--isoform-X2-1a_2-P-clavata	PREDICTED: neprilysin-11 isoform X2		DN10125_c0_g18_i1	2	4	43	1.7	Y
Matrix-metalloproteinase-14-like-2a-P-clavata	PREDICTED: matrix metalloproteinase-14 isoform	*D. quadriceps*	DN21699_c0_g1_i1	2	6	41	1.9	Y
Protein-5NUC-1a-P-clavata	PREDICTED: protein 5NUC-like	*Wasmannia auropunctata*	DN10097_c1_g11_i1	1	2	37	25.2	N
Icarapin-1a-P-clavata	PREDICTED: icarapin-like	*D. quadriceps*	DN9969_c1_g1_i4	1	7	32	969	N
Cytosolic-dipeptidase-1a-P-clavata	PREDICTED: cytosolic non-specific dipeptidase	*D. quadriceps*	DN9768_c0_g1_i2	1	2	30	1.8	Y
Phenoloxidase-2-like-1a-P-clavata	PREDICTED: phenoloxidase 2	*S. invicta*	DN15311_c0_g1_i1	1	4	24	3.2	Y

**Table 4:** Predicted *P. clavata* peptides conforming to ICK or conotoxin cysteine framework I frameworks. This table shows peptides identified using Tox|Note and their corresponding expression level indicated by TPM. However, peptides containing eight or more cysteines that conform to the ICK framework typically have four cysteines after the “CC” doublet, where two of the cysteines turn the fourth loop into a disulfide-stabilised hairpin. *P. clavata* peptides with eight or more cysteines may therefore represent putatively novel cysteine-rich scaffolds.

Number of Cysteines	Scaffold	Mature Peptide Length	TPM
4	x_8_CCx_1_Cx_19_Cx_5_	37	772
	x_1_CCx_34_Cx_1_Cx_1_	41	18.4
	x_5_CCx_1_Cx_16_Cx_14_	40	16.5
	CCx_23_Cx_7_Cx_6_	40	13
	x_14_CCx_11_Cx_7_Cx_33_	69	10.8
	CCx_1_Cx_27_Cx_12_	44	7.1
	x_3_CCx_6_Cx_15_Cx_9_	37	2.2
	x_6_CCx_7_Cx_11_Cx_8_	36	0.76
	x_8_CCx_5_Cx_12_Cx_6_	35	0.3
6	Cx_6_Cx_5_CCx_4_Cx_6_Cx_29_	56	43.8
	x_18_Cx_6_Cx_3_CC_8_Cx_24_Cx_7_	72	9.3
	x_7_Cx_8_Cx_21_CCx_5_Cx_22_Cx_20_	89	5.9
	Cx_6_Cx_5_CCx_4_Cx_6_Cx_29_	56	3.9
	x_8_Cx_6_Cx_5_CCx_4_Cx_6_Cx_25_	60	3.4
	x_8_Cx_6_Cx_5_CCx_4_Cx_6_Cx_7_	42	2.6
8	x_14_Cx_9_Cx_3_Cx_5_Cx_5_CCx_3_Cx_4_Cx_26_	77	1.76
10	x_10_Cx_5_Cx_4_CCx_11_Cx_9_Cx_28_Cx_1_Cx_5_Cx_6_Cx_1_	90	12.67

## Supplementary Materials

The following are available online at https://www.mdpi.com/2072-6651/12/5/324/s1, Figure S1: Distribution of protein hits to different hymenopteran species, Figure S2: δ-Paraponeritoxin-Pc1e LC-MS/MS coverage, Figure S3: Isolated *P. clavata* poneratoxin MSMS match to δ-paraponeritoxin-Pc1e_1 contig, Figure S4: Amino acid sequence alignment of ω-conotoxin-like contigs, Figure S5: Alignment of hyaluronidase-like proteins from *P. clavata* and other ant species, Figure S6: Alignment of icarapin-like proteins, Figure S7: Amino acid alignment of arginine kinase transcripts, Figure S8: Alignment of serine proteases from *P. clavata* and other insect species, Figure S9: Summary of the *P. clavata* combined proteome/ transcriptome methodology, Figure S10: Gene ontology classification of contigs with BLASTx hits, Figure S11: Peptide toxin nomenclature system using a spider venom peptide example, Figure S12: Proposed protein toxin nomenclature system using an ant venom protein example, Table S1: Toxin keyword search list. Supplementary file S1: toxins identified by BLASTx along with the TPM values, Supplementary file S2: an excel worksheet containing all protein hits from the proteome investigation using the transcriptome database.

## References

[1] Schendel V., Rash L.D., Jenner R.A., Undheim E.A.B. (2019). The diversity of venom: The importance of behavior and venom system morphology in understanding its ecology and evolution. Toxins.

[2] Robinson S.D., Undheim E.A.B., Ueberheide B., King G.F. (2017). Venom peptides as therapeutics: Advances, challenges and the future of venom-peptide discovery. Expert Rev. Proteom..

[3] Smith J.J., Undheim E.A.B. (2018). True lies: Using proteomics to assess the accuracy of transcriptome-based venomics in centipedes uncovers false positives and reveals startling intraspecific variation in *Scolopendra subspinipes*. Toxins.

[4] Piek T., Duval A., Hue B., Karst H., Lapied B., Mantel P., Nakajima T., Pelhate M., Schmidt J.O. (1991). Poneratoxin, a novel peptide neurotoxin from the venom of the ant, *Paraponera clavata*. Comp. Biochem. Physiol. Part C Comp. Pharm..

[5] Piek T., Hue B., Mantel P., Nakajima T., Schmidt J.O. (1991). Pharmacological characterization and chemical fractionation of the venom of the ponerine ant, *Paraponera clavata* (F.). Comp. Biochem. Physiol. Part C Comp. Pharm..

[6] Duval A., Malécot C.O., Pelhate M., Piek T. (1992). Poneratoxin, a new toxin from an ant venom, reveals an interconversion between two gating modes of the Na channels in frog skeletal muscle fibres. Pflügers Arch..

[7] Hendrich A.B., Mozrzymas J.W., Konopińska D., Scuka M. (2001). The effect of poneratoxin on neuromuscular transmission in the rat diaphragm. Cell. Mol. Biol. Lett..

[8] Szolajska E., Poznanski J., Ferber M.L., Michalik J., Gout E., Fender P., Bailly I., Dublet B., Chroboczek J. (2004). Poneratoxin, a neurotoxin from ant venom. Structure and expression in insect cells and construction of a bio-insecticide. Eur. J. Biochem..

[9] Rykaczewska-Czerwinska M., Radosz A., Konopinska D., Wrobel M., Plech A. (2008). Antinociceptive effect of poneratoxin [PoTX] in rats. Pestycydy.

[10] Johnson S.R., Rikli H.G., Schmidt J.O., Evans M.S. (2017). A reexamination of poneratoxin from the venom of the bullet ant *Paraponera clavata*. Peptides.

[11] Aili S.R., Touchard A., Petitclerc F., Dejean A., Orivel J., Padula M.P., Escoubas P., Nicholson G.M. (2017). Combined peptidomic and proteomic analysis of electrically stimulated and manually dissected venom from the south American bullet ant *Paraponera clavata*. J. Proteome Res..

[12] Aili S.R., Touchard A., Koh J.M.S., Dejean A., Orivel J., Padula M.P., Escoubas P., Nicholson G.M. (2016). Comparisons of protein and peptide complexity in poneroid and formicoid ant venoms. J. Proteome Res..

[13] Haas B.J., Papanicolaou A., Yassour M., Grabherr M., Blood P.D., Bowden J., Couger M.B., Eccles D., Li B., Lieber M. (2013). *De novo* transcript sequence reconstruction from RNA-seq using the trinity platform for reference generation and analysis. Nat. Protoc..

[14] Altschul S.F., Madden T.L., Schaffer A.A., Zhang J., Zhang Z., Miller W., Lipman D.J. (1997). Gapped BLAST and PSI-BLAST: A new generation of protein database search programs. Nucleic Acids Res..

[15] Pineda S.S., Chaumeil P.-A., Kunert A., Kaas Q., Thang M.W., Li L., Nuhn M., Herzig V., Saez N.J., Cristofori-Armstrong B. (2017). ArachnoServer 3.0: An online resource for automated discovery, analysis and annotation of spider toxins. Bioinformatics.

[16] Graudins A., Little M.J., Pineda S.S., Hains P.G., King G.F., Broady K.W., Nicholson G.M. (2012). Cloning and activity of a novel alpha-latrotoxin from red-back spider venom. Biochem. Pharm..

[17] De Junqueira-de-Azevedo L.M.I., Pertinhez T., Spisni A., Carreño F.R., Farah C.S., Ho P.L. (2003). Cloning and expression of calglandulin, a new EF-hand protein from the venom glands of *Bothrops insularis* snake in E. coli. Biochim. Biophys. Acta BBA Proteins Proteom..

[18] Petersen T.N., Brunak S., von Heijne G., Nielsen H. (2011). SignalP 4.0: Discriminating signal peptides from transmembrane regions. Nat. Methods.

[19] Piek T. (1990). Neurotoxins from venoms of the hymenoptera-twenty-five years of research in Amsterdam. Comp. Biochem. Physiol. Part C Comp. Pharm..

[20] Kumar S., Stecher G., Tamura K. (2016). MEGA7: Molecular evolutionary genetics analysis version 7.0 for bigger datasets. Mol. Biol. Evol..

[21] McCleskey E., Fox A., Feldman D., Cruz L., Olivera B., Tsien R., Yoshikami D. (1987). Omega-conotoxin: Direct and persistent blockade of specific types of calcium channels in neurons but not muscle. Proc. Natl. Acad. Sci. USA.

[22] Gutiérrez J.M., Lomonte B. (2013). Phospholipases A2: Unveiling the secrets of a functionally versatile group of snake venom toxins. Toxicon.

[23] Scott D.L., Otwinowski Z., Gelb M.H., Sigler P.B. (1990). Crystal structure of bee-venom phospholipase A2 in a complex with a transition-state analogue. Science.

[24] Six D.A., Dennis E.A. (2000). The expanding superfamily of phospholipase A2 enzymes: Classification and characterization. Biochim. Biophys. Acta BBA Mol. Cell Biol. Lipids.

[25] Valentin E., Ghomashchi F., Gelb M.H., Lazdunski M., Lambeau G. (2000). Novel human secreted phospholipase A2 with homology to the group III bee venom enzyme. J. Biol. Chem..

[26] Kuchler K., Gmachl M., Sippl M.J., Kreil G. (1989). Analysis of the cDNA for phospholipase A2 from honeybee venom glands. Eur. J. Biochem..

[27] Bouzid W., Verdenaud M., Klopp C., Ducancel F., Noirot C., Vétillard A. (2014). De novo sequencing and transcriptome analysis for *Tetramorium bicarinatum*: A comprehensive venom gland transcriptome analysis from an ant species. BMC Genom..

[28] White S.P., Scott D.L., Otwinowski Z., Gelb M.H., Sigler P.B. (1990). Crystal structure of cobra-venom phospholipase A_2_ in a complex with a transition-state analogue. Science.

[29] Kini R.M. (2005). Structure-function relationships and mechanism of anticoagulant phospholipase A 2 enzymes from snake venoms. Toxicon.

[30] Chioato L., Ward R.J. (2003). Mapping structural determinants of biological activities in snake venom phospholipases A2 by sequence analysis and site directed mutagenesis. Toxicon.

[31] Marković-Housley Z., Miglierini G., Soldatova L., Rizkallah P.J., Müller U., Schirmer T. (2000). Crystal structure of hyaluronidase, a major allergen of bee venom. Structure.

[32] Peiren N., de Graaf D.C., Brunain M., Bridts C.H., Ebo D.G., Stevens W.J., Jacobs F.J. (2006). Molecular cloning and expression of icarapin, a novel IgE-binding bee venom protein. FEBS Lett..

[33] Baek J.H., Lee S.H. (2010). Identification and characterization of venom proteins of two solitary wasps, *Eumenes pomiformis* and *Orancistrocerus drewseni*. Toxicon.

[34] Freeman T.M., Tracy J.M. (2017). Stinging Insect Allergy: A Clinician’s Guide.

[35] Schmidt J.O. (2016). The Sting of the Wild.

[36] Torres A.F.C., Huang C., Chong C.-M., Leung S.W., Prieto-da-Silva A.R.B., Havt A., Quinet Y.P., Martins A.M.C., Lee S.M.Y., Rádis-Baptista G. (2014). Transcriptome analysis in venom gland of the predatory giant ant *Dinoponera quadriceps*: Insights into the polypeptide toxin arsenal of Hymenopterans. PLoS ONE.

[37] Ponce D., Brinkman D.L., Potriquet J., Mulvenna J. (2016). Tentacle transcriptome and venom proteome of the pacific sea nettle, *Chrysaora fuscescens* (*Cnidaria: Scyphozoa*). Toxins.

[38] Elsik C.G., Tayal A., Diesh C.M., Unni D.R., Emery M.L., Nguyen H.N., Hagen D.E. (2016). Hymenoptera Genome Database: Integrating genome annotations in HymenopteraMine. Nucleic Acids Res..

[39] Aili S.R., Touchard A., Escoubas P., Padula M.P., Orivel J., Dejean A., Nicholson G.M. (2014). Diversity of peptide toxins from stinging ant venoms. Toxicon.

[40] Moreau C.S., Bell C.D., Vila R., Archibald S.B., Pierce N.E. (2006). Phylogeny of the ants: Diversification in the age of angiosperms. Science.

[41] Robinson S.D., Mueller A., Clayton D., Starobova H., Hamilton B.R., Payne R.J., Vetter I., King G.F., Undheim E.A. (2018). A comprehensive portrait of the venom of the giant red bull ant, *Myrmecia gulosa*, reveals a hyperdiverse hymenopteran toxin gene family. Sci. Adv..

[42] Kozlov S., Malyavka A., McCutchen B., Lu A., Schepers E., Herrmann R., Grishin E. (2005). A novel strategy for the identification of toxin-like structures in spider venom. Proteins Struct. Funct. Bioinform..

[43] Wong E.S., Hardy M.C., Wood D., Bailey T., King G.F. (2013). SVM-based prediction of propeptide cleavage sites in spider toxins identifies toxin innovation in an Australian tarantula. PLoS ONE.

[44] Mouls L., Subra G., Aubagnac J.L., Martinez J., Enjalbal C. (2006). Tandem mass spectrometry of amidated peptides. J. Mass Spectrom..

[45] Labella C., Kanawati B., Vogel H., Schmitt-Kopplin P., Laurino S., Bianco G., Falabella P. (2015). Identification of two arginine kinase forms of endoparasitoid *Leptomastix dactylopii* venom by bottom up-sequence tag approach. J. Mass Spectrom..

[46] Yamamoto T., Arimoto H., Kinumi T., Oba Y., Uemura D. (2007). Identification of proteins from venom of the paralytic spider wasp, *Cyphononyx dorsalis*. Insect Biochem. Mol. Biol..

[47] Choo Y.M., Lee K.S., Yoon H.J., Kim B.Y., Sohn M.R., Roh J.Y., Je Y.H., Kim N.J., Kim I., Woo S.D. (2010). Dual function of a bee venom serine protease: Prophenoloxidase-activating factor in arthropods and fibrin(ogen)olytic enzyme in mammals. PLoS ONE.

[48] Matsui T., Fujimura Y., Titani K. (2000). Snake venom proteases affecting hemostasis and thrombosis. Biochim. Biophys. Acta BBA Protein Struct. Mol. Enzymol..

[49] Jiang H., Kanost M.R. (2000). The clip-domain family of serine proteinases in arthropods. Insect Biochem. Mol. Biol..

[50] Winningham K.M., Fitch C.D., Schmidt M., Hoffman D.R. (2004). Hymenoptera venom protease allergens. J. Allergy Clin. Immunol..

[51] Laurino S., Grossi G., Pucci P., Flagiello A., Bufo S.A., Bianco G., Salvia R., Vinson S.B., Vogel H., Falabella P. (2016). Identification of major *Toxoneuron nigriceps* venom proteins using an integrated transcriptomic/proteomic approach. Insect Biochem. Mol. Biol..

[52] Asgari S., Zhang G., Zareie R., Schmidt O. (2003). A serine proteinase homolog venom protein from an endoparasitoid wasp inhibits melanization of the host hemolymph. Insect Biochem. Mol. Biol..

[53] Ross J., Jiang H., Kanost M.R., Wang Y. (2003). Serine proteases and their homologs in the *Drosophila melanogaster* genome: An initial analysis of sequence conservation and phylogenetic relationships. Gene.

[54] Piao S., Song Y.L., Kim J.H., Park S.Y., Park J.W., Lee B.L., Oh B.H., Ha N.C. (2005). Crystal structure of a clip-domain serine protease and functional roles of the clip domains. EMBO J..

[55] Kazuma K., Masuko K., Konno K., Inagaki H. (2017). Combined venom gland transcriptomic and venom peptidomic analysis of the predatory ant *Odontomachus monticola*. Toxins.

[56] Luna-Ramírez K., Quintero-Hernández V., Juárez-González V.R., Possani L.D. (2015). Whole transcriptome of the venom gland from *Urodacus yaschenkoi* scorpion. PLoS ONE.

[57] Kini R.M. (2003). Excitement ahead: Structure, function and mechanism of snake venom phospholipase a 2 enzymes. Toxicon.

[58] Jacomini D.L.J., Pereira F.D.C., dos Santos Pinto J.R.A., dos Santos L.D., da Silva Neto A.J., Giratto D.T., Palma M.S., de Lima Zollner R., Braga M.R.B. (2013). Hyaluronidase from the venom of the social wasp *Polybia paulista* (*Hymenoptera, Vespidae*): Cloning, structural modeling, purification, and immunological analysis. Toxicon.

[59] Jin C., Focke M., Léonard R., Jarisch R., Altmann F., Hemmer W. (2010). Reassessing the role of hyaluronidase in yellow jacket venom allergy. J. Allergy Clin. Immunol..

[60] Hoffman D.R. (2006). Hymenoptera venom allergens. Clin. Rev. Allergy Immunol..

[61] Terlau H., Olivera B.M. (2004). Conus venoms: A rich source of novel ion channel-targeted peptides. Physiol. Rev..

[62] King G.F., Gentz M.C., Escoubas P., Nicholson G.M. (2008). A rational nomenclature for naming peptide toxins from spiders and other venomous animals. Toxicon.

[63] Zobel-Thropp P.A., Kerins A.E., Binford G.J. (2012). Sphingomyelinase D in sicariid spider venom is a potent insecticidal toxin. Toxicon.

[64] Binford G.J., Cordes M.H., Wells M.A. (2005). Sphingomyelinase D from venoms of *Loxosceles* spiders: Evolutionary insights from cDNA sequences and gene structure. Toxicon.

[65] Ueberheide B.M., Fenyö D., Alewood P.F., Chait B.T. (2009). Rapid sensitive analysis of cysteine rich peptide venom components. Proc. Natl. Acad. Sci. USA.

[66] Bloch G., Cohen M. (2014). The expression and phylogenetics of the inhibitor cysteine knot peptide OCLP1 in the honey bee *Apis mellifera*. J. Insect Physiol..

[67] Arias H.R., Blanton M.P. (2000). α-Conotoxins. Int. J. Biochem. Cell Biol..

[68] Dutertre S., Jin A.H., Kaas Q., Jones A., Alewood P.F., Lewis R.J. (2013). Deep venomics reveals the mechanism for expanded peptide diversity in cone snail venom. Mol. Cell. Proteom..

[69] Lader E.S. (2012). Methods and Reagents for Preserving Rna in Cell and Tissue Samples.

[70] Andrews S. (2011). FastQC: A quality control tool for high throughput sequence data.

[71] Lohse M., Bolger A.M., Nagel A., Fernie A.R., Lunn J.E., Stitt M., Usadel B. (2012). R obi NA: A user-friendly, integrated software solution for RNA-Seq-based transcriptomics. Nucleic Acids Res..

[72] Langmead B., Salzberg S.L. (2012). Fast gapped-read alignment with Bowtie 2. Nat. Methods.

[73] Li B., Dewey C.N. (2011). RSEM: Accurate transcript quantification from RNA-Seq data with or without a reference genome. BMC Bioinform..

[74] Consortium U. (2008). The universal protein resource (UniProt). Nucleic Acids Res..

[75] Huntley R.P., Sawford T., Mutowo-Meullenet P., Shypitsyna A., Bonilla C., Martin M.J., O’Donovan C. (2015). The GOA database: Gene ontology annotation updates for 2015. Nucleic Acids Res..

[76] Grabherr M.G., Haas B.J., Yassour M., Levin J.Z., Thompson D.a., Amit I., Adiconis X., Fan L., Raychowdhury R., Zeng Q. (2011). Full-length transcriptome assembly from RNA-Seq data without a reference genome. Nat. Biotechnol..

[77] Zhang J., Xin L., Shan B., Chen W., Xie M., Yuen D., Zhang W., Zhang Z., Lajoie G.A., Ma B. (2012). PEAKS DB: De novo sequencing assisted database search for sensitive and accurate peptide identification. Mol. Cell. Proteom..

[78] Touchard A., Aili S., Fox E., Escoubas P., Orivel J., Nicholson G., Dejean A. (2016). The biochemical toxin arsenal from ant venoms. Toxins.

