# Supplementary material for: An Integrated Proteomic and Transcriptomic Analysis Reveals the Venom Complexity of the Bullet Ant Paraponera clavata
Source: Toxins (Basel). 2020 May 14;12(5):324. doi: 10.3390/toxins12050324 (PMC7290781; doi:10.3390/toxins12050324)
Supplement: Supplementary file 1 [file toxins-12-00324-s001.zip › supplementary materials/toxins-772574 supplementary for final.docx]

Supplementary Materials: An Integrated Proteomic and Transcriptomic Analysis Reveals the Venom Complexity of the Bullet Ant *Paraponera clavata*

Samira R. Aili, Axel Touchard, Regan Hayward, Samuel D. Robinson, Sandy S. Pineda,
Hadrien Lalagüe, Mrinalini, Irina Vetter, Eivind A. B. Undheim, R. Manjunatha Kini, Pierre Escoubas, Matthew P. Padula, Garry Myers and Graham M. Nicholson


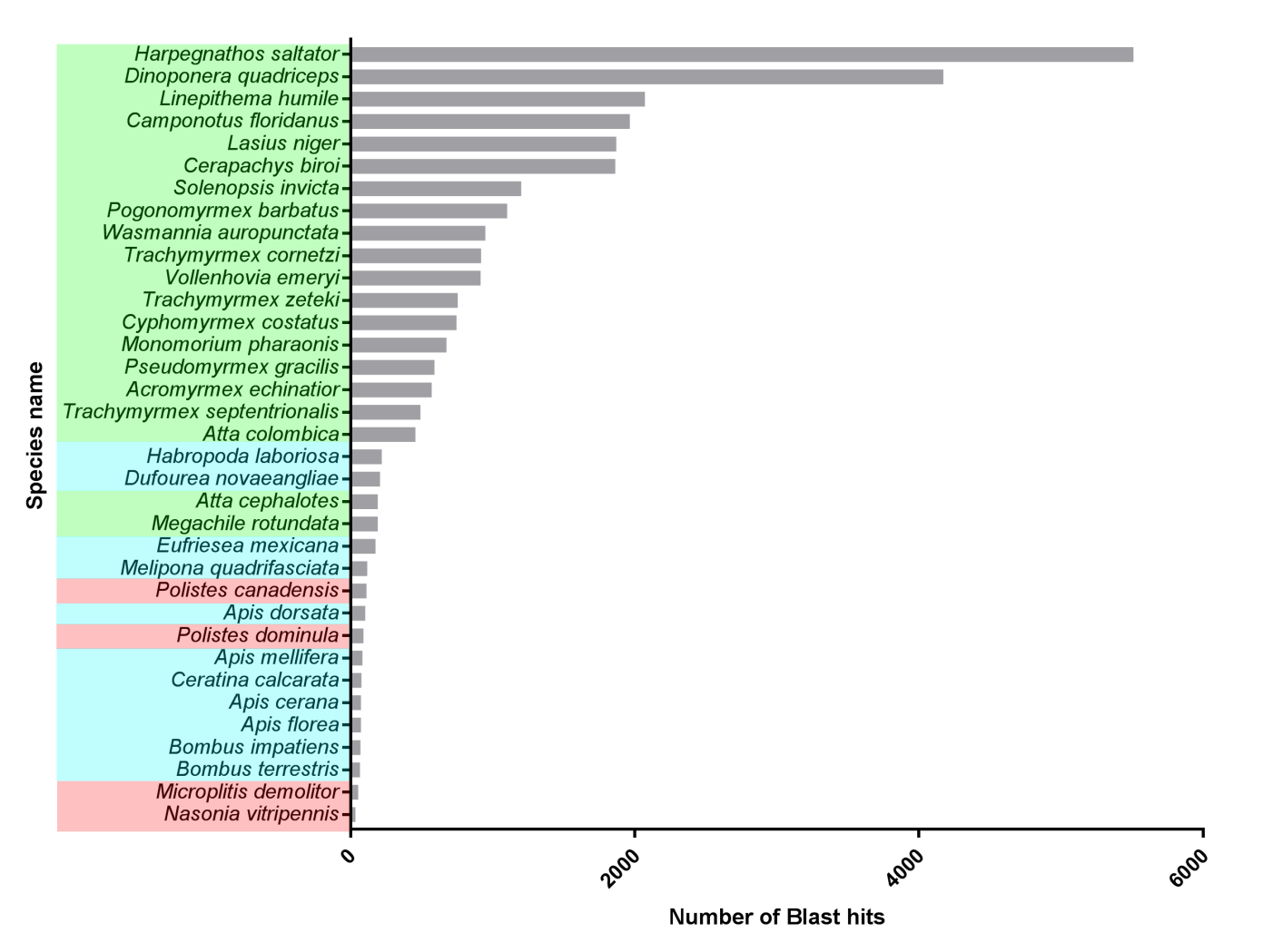


**Figure S1.** Distribution of protein hits to different hymenopteran species. Graph shows the number of protein hits in the top 35 species matched by BLASTx, highlighted regions indicate matches to ants (green), bees (cyan) and wasps (pink). The most prevalent protein/ peptide hits were to the ant *Harpegnathos saltator.*


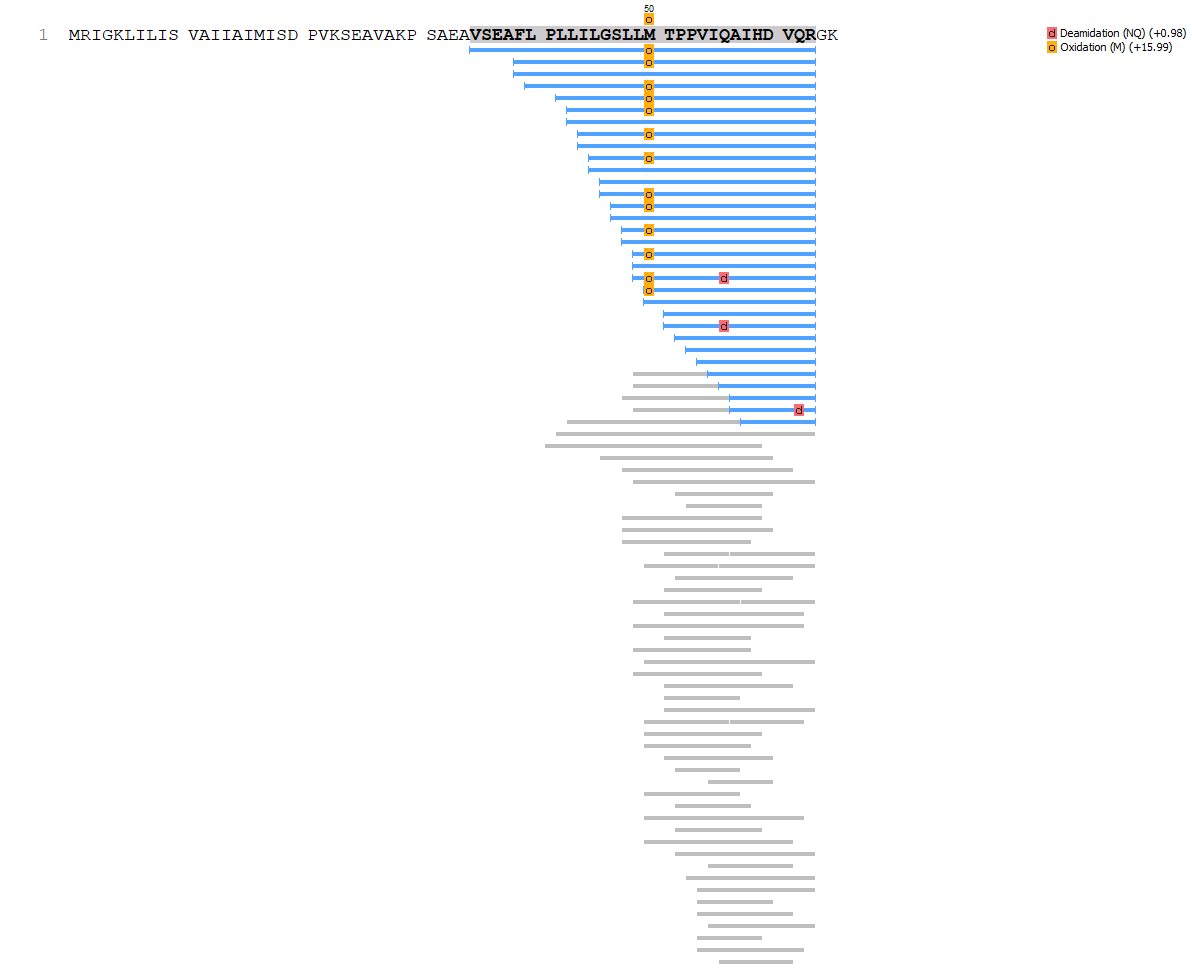


**Figure S2.** δ-Paraponeritoxin-Pc1e LC-MS/MS coverage. .Figure shows translated δ-paraponeritoxin-Pc1e_1 transcript coverage obtained from the shotgun mass spectrometry search result from PEAKS. Blue lines indicate an identified peptide sequence and grey bars indicate a *de novo* only tag match. Yellow ‘o’ indicates oxidation of methionine and red ‘d’ indicates deamidation.


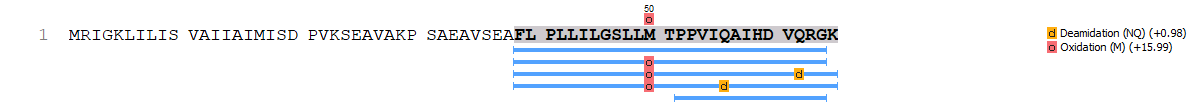


**Figure S3.** Isolated *P. clavata* poneratoxin MSMS match to δ-paraponeritoxin-Pc1e_1 contig**.** Peptide was isolated using RP-HPLC and used in neuronal activity assay.


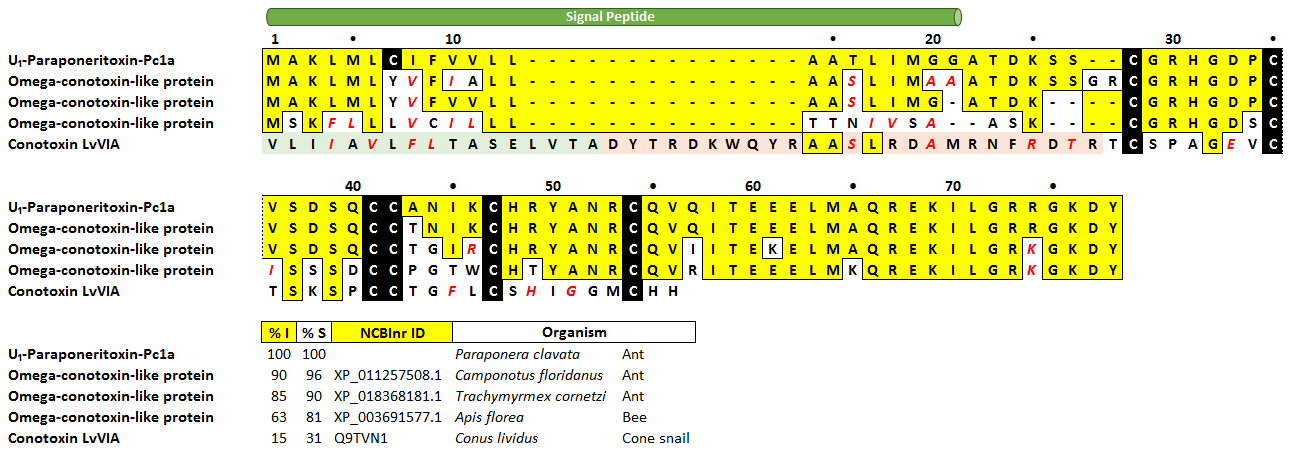


**Figure S4.** Amino acid sequence alignment of ω-conotoxin-like contigs. The Figure shows alignment of *P. clavata* contigs and proteins from the ant species *C. floridanus* and *T. cornetzi,* the bee *Apis florea* and the marine cone snail *Conus lividus.* The predicted signal peptide for all sequences, except conotoxin LvVIA, is indicated by a green bar above the sequences. The signal peptide for conotoxin LvVIA is indicated by light green shading, while orange shading indicates the propeptide. Identical residues are boxed in yellow while conservative substitutions are shown in red italic text. Cysteines are boxed in black. Gaps were introduced to optimize the alignments. Percentage identity (%I) is relative to U_1_-paraponeritoxin-Pc1a, while percentage similarity (%S) includes conservatively substituted residues.

**
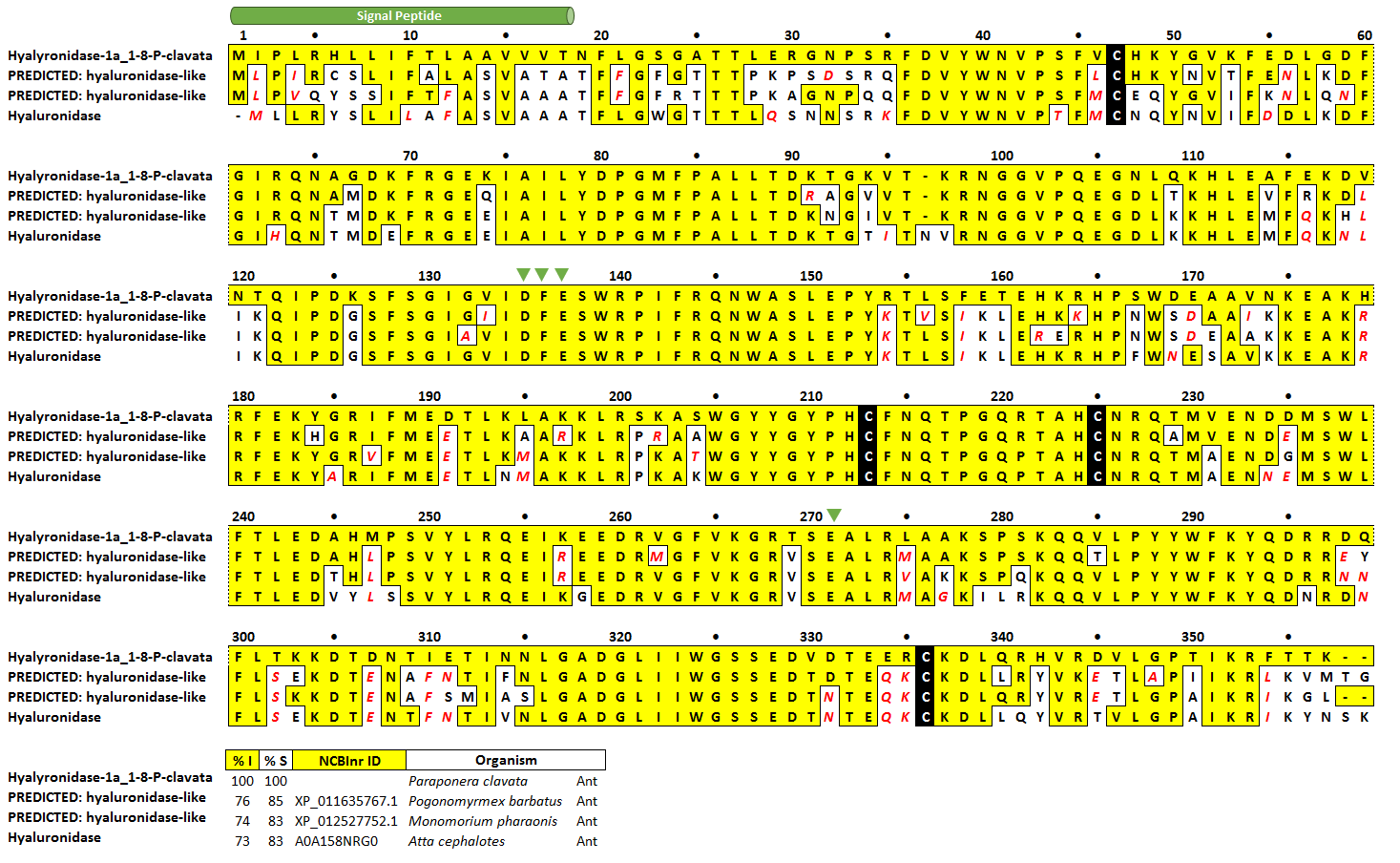
**

**Figure S5.** Alignment of hyaluronidase-like proteins from *P. clavata* and other ant species. The Figure shows the alignment of *P. clavata* transcripts (hyaluronidase-1a_1-P-clavata through to ‘-1a_8’ identical ORFs) and hyaluronidase proteins from the ants *Pogonomyrmex barbatus*, *Monomorium pharaonis* and *Atta cephalotes*. A signal peptide was predicted for all sequences except for that of *M. pharonis* (indicated by a green bar above the sequences). All signal peptides were from amino acid 1–18, except that of *A. cephalotes* which was from 1-17. Identical residues in the peptide sequences are boxed in yellow while conservative substitutions are shown in red italic text. Cysteines are boxed in black. Gaps were introduced to optimize the alignments. Percentage identity (%I) is relative to hyaluronidase-1a_1-P-clavata, while percentage similarity (%S) includes conservatively substituted residues. Green arrowheads denote the active-site residues Asp, Phe, Glu and Glu of bee venom hyaluronidase {Marković-Housley, 2000 #899}.


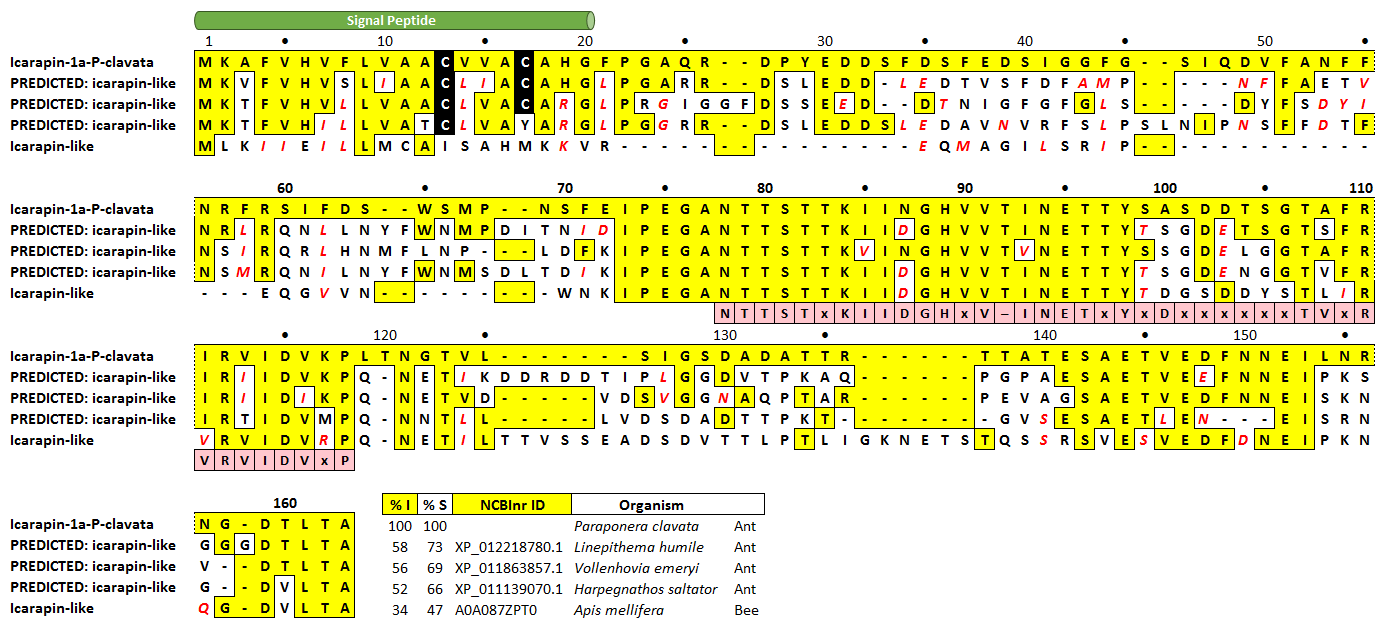


**Figure S6.** Alignment of icarpin-like proteins. The figure shows the alignment of the *P. clavata* transcript with homologous icarapin-like proteins from the ant species *Linepithema humile*, *Vollenhovia emeryi*, *Harpegnathos saltator* and the bee *Apis mellifera.* Predicted signal peptide (green bar) is indicated above the sequences, it was the same size for all sequences except *A. mellifera’s* which ends at amino acid 17. Identical residues in the peptide sequences are boxed in yellow while conservative substitutions are shown in red italic text. Cysteines are boxed in black. Gaps were introduced to optimise the alignments. Boxed residues in pink indicate the conserved icarapin residues described by Pieren (2006) {Peiren, 2006 #888}. Percentage identity (%I) is relative to icarapin-1a-P-clavata, while percentage similarity (%S) includes conservatively substituted residues.


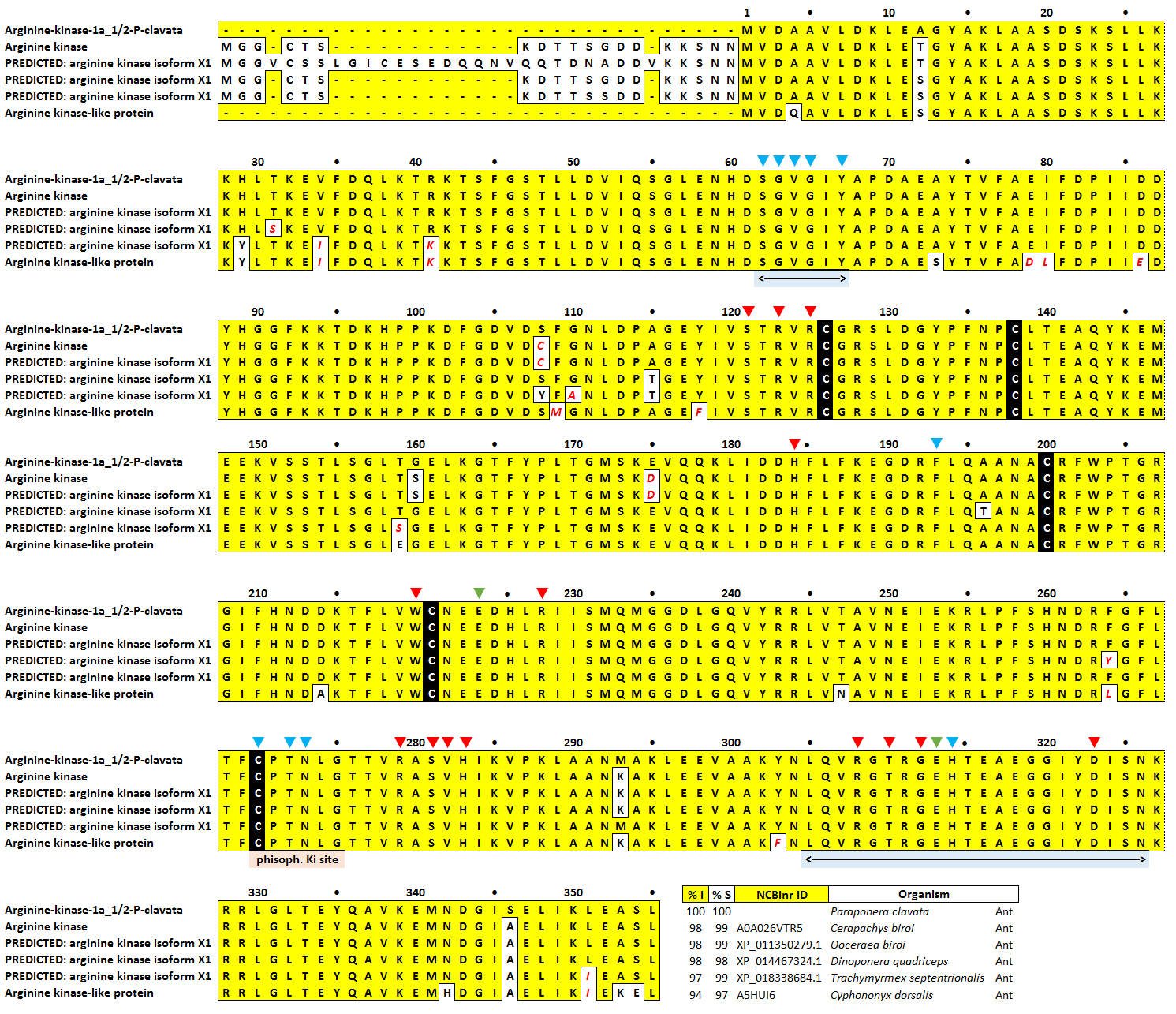


**Figure S7.** Amino acid alignment of arginine kinase transcripts. Alignment of *P. clavata* arginine-kinase-1a-P-clavata to proteins from the ant species *Cerapachys biroi*, *Ooceraea biroi*, *Dinoponera quadriceps*, *Trachymyrmex septentrionalis* and *Cyphononyx dorsalis*. Identical residues in the peptide sequences are boxed in yellow while conservative substitutions are shown in red italic text. Cysteines are boxed in black. Gaps were introduced to optimize the alignments. Percentage identity (%I) is relative to arginine-kinase-1a-P-clavata, while percentage similarity (%S) includes conservatively substituted residues. Blue triangles indicate amino acid residues implicated in arginine binding, red triangles show residues important for ATP binding and green triangles indicate residues important for catalytic binding {Strong, 1995 #927}. Blue boxed regions with ’<->’ indicates the specificity loops {Baek, 2010 #885}. Finally, the orange boxed region labelled “phosphagen kinase” represents the phosphagen kinase site {Strong, 1995 #927}.

**
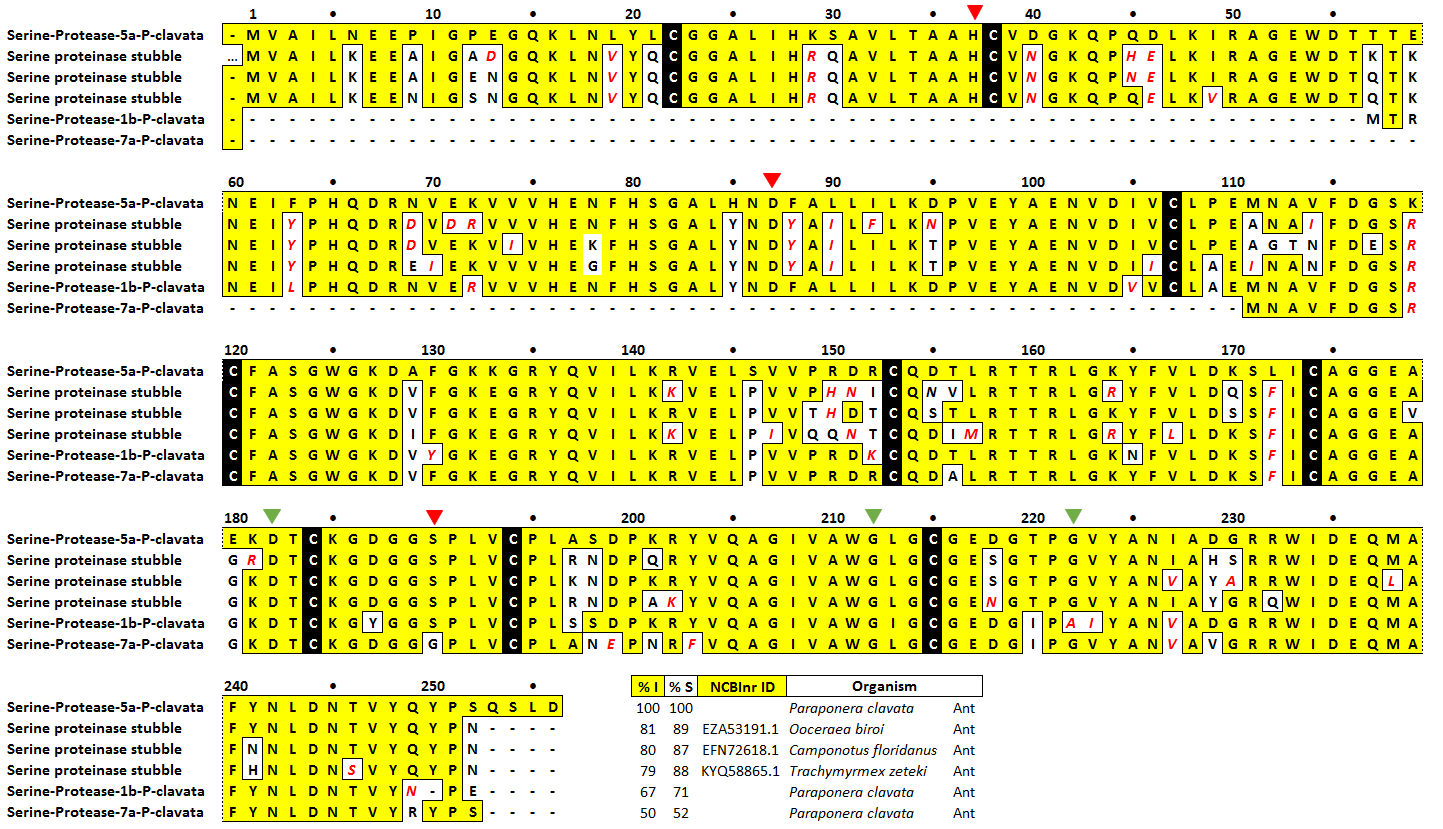
**

**Figure S8.** Alignment of serine proteases from *P. clavata* and other insect species. The Figure shows alignment of three *P. clavata* transcripts (serine-protease-5a-P-clavata, serine-protease-1b-P-clavata and serine-protease-7a-P-clavata) that have homology to other serine protease-like proteins from the ant species *Ooceraea biroi*, *Camponotus floridanus* and *Trachymyrmex zeteki.* The only sequence with a predicted signal peptide was that of *O. biroi* (not shown, but started at amino acid 174). Identical residues in the peptide sequences are boxed in yellow whilst conservative substitutions are shown in red italic text. Cysteines are boxed in black. Gaps were introduced to optimize the alignments. Solid green triangles indicate the important residues for the specificity pocket {Jiang, 2000 #872;Piao, 2005 #874}. Solid red triangles indicate the catalytic triad (His, Asp, Ser) {Ross, 2003 #875;Choo, 2010 #877}. Percentage identity (%I) is relative to serine-protease-5a-P-clavata, while percentage similarity (%S) includes conservatively substituted residues.


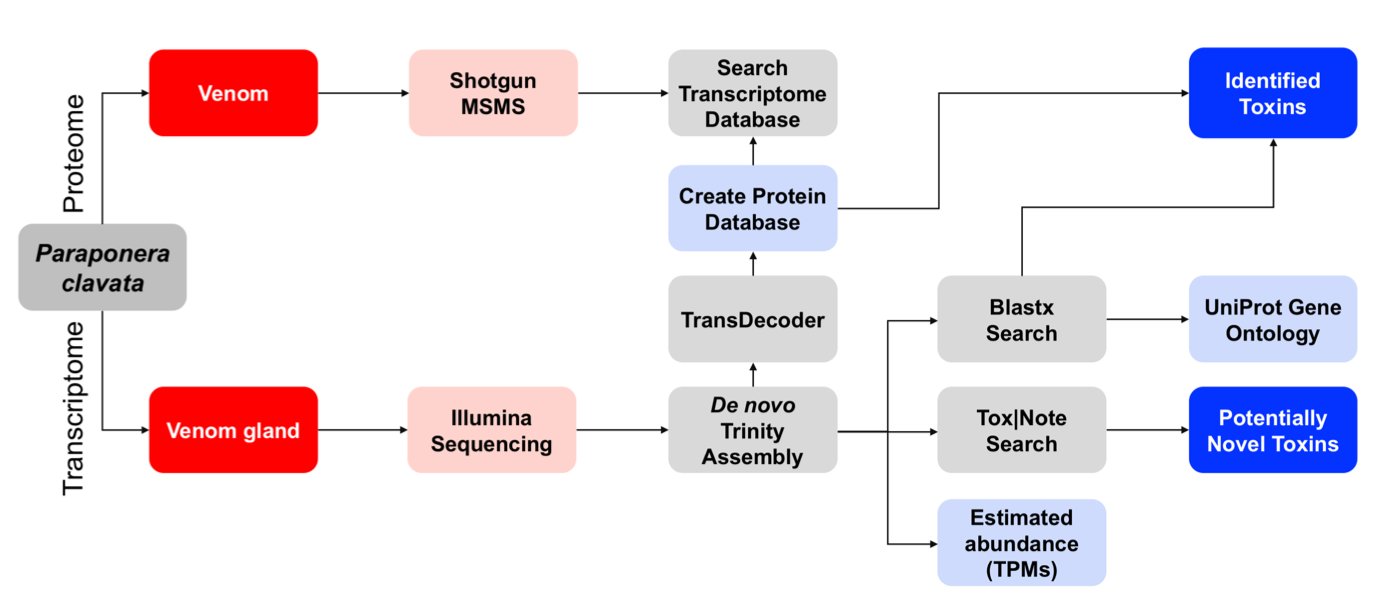


**Figure S9.** Summary of the *P. clavata* combined proteome/ transcriptome methodology.Flow chart outlines the main steps undertaken in the present investigation using an integrated proteomic and transcriptomic methodology.


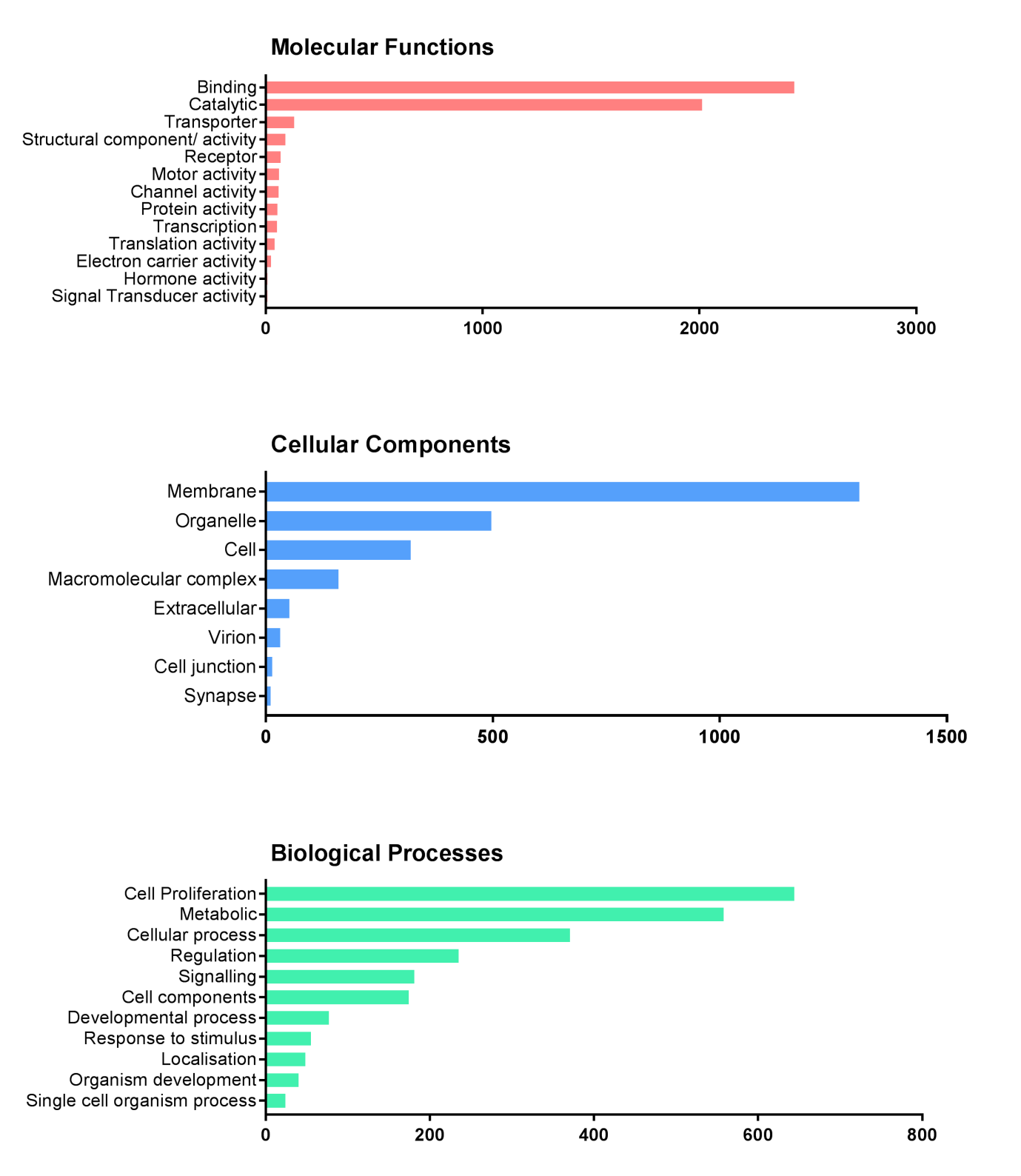


**Figure S10.** Gene Ontology classification of contigs with BLASTx hits. Figure shows the distribution of hits to each of the categories – molecular function, biological processes and cellular processes. Results were obtained by searching accessions obtained by BLASTx in the UniProtKB Retrieve/ID mapping extension (http://[www.uniprot.org/)](http://www.uniprot.org/)).


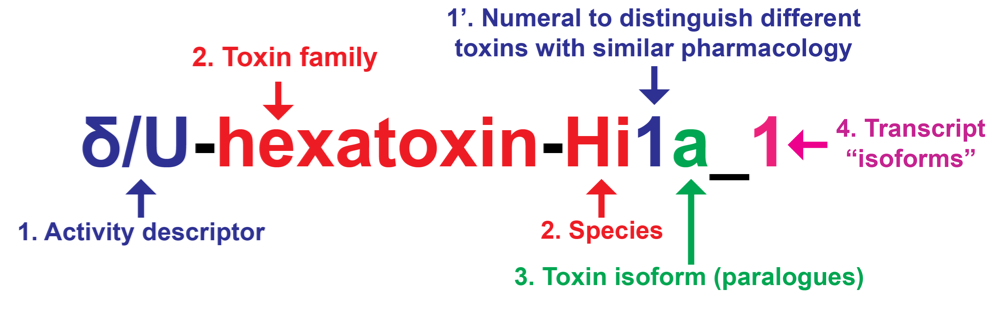


**Figure S11.** Peptide toxin nomenclature system using a spider venom peptide example. The toxin name is divided into three parts that describe the toxin’s activity (blue), biological source (red), and relationship to other toxins (green/purple). The subtype descriptor should be based on IUPHAR-recommended nomenclature for channels and receptors. Figure adapted from King et al. (2008) {King, 2008 #848}.


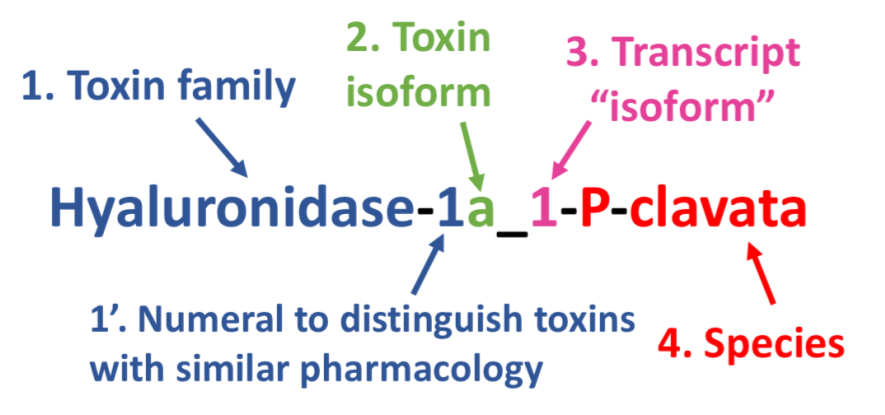


**Figure S12.** Proposed protein toxin nomenclature system using an ant venom protein example. The toxin name is divided into three parts that describe the toxin’s family (blue), biological source (red), and relationship to other toxins (green/purple).

**Table S1.** Toxin keyword search list. Table shows a list of common toxin protein names previously used to describe venom toxins.

| Common Toxin Protein Names Ectatomin | | | | |
| --- | --- | --- | --- | --- |
| Acetylcholinesterase | **Bicarinalin** | **Ectatomin** | **Mastoparan** | **Ponericin** |
| ACh | Calsyntenin | Esterase | Metalloendopeptidase | Serine prot |
| Acid Oxidase | Carboxypeptidase | Haemorrhagic | Metalloprotease/peptidase | Serine Protease/Proteinase |
| Acid phosphatase | Cholinesterase | Hyaluronidase | Metalloproteinase | Serpin (serine protease inhibitor) |
| ADAM | Coagulation | Icarapin | Neprilysin | SNTX (Stonustoxin) |
| Alkaline phosphatase | Conotoxin | Kinin | Phospholipase | Sphingomyelin D |
| Allergen | CRISP/ cysteine rich secretory protein | LAAO amino oxidase | Pilosulin | STX (Shiga Toxin) |
| Analgesic | Defensin | Lectin | PLA | Toxin/ Tx |
| Antigen | Dipeptidyl peptidase | Leucine-rich domain | PLB | VEGF, vascular |
| Arginine kinase | Disintegrin | Lipase | PLD | Venom |
